# Graphene oxide elicits microbiome-dependent type 2 immune responses via the aryl hydrocarbon receptor

**DOI:** 10.1038/s41565-022-01260-8

**Published:** 2022-12-12

**Authors:** Guotao Peng, Hanna M. Sinkko, Harri Alenius, Neus Lozano, Kostas Kostarelos, Lars Bräutigam, Bengt Fadeel

**Affiliations:** 1 Institute of Environmental Medicine, Karolinska Institutet, Stockholm, Sweden; 2 Human Microbiome Research Program (HUMI), University of Helsinki, Helsinki, Finland; 3 Catalan Institute of Nanoscience and Nanotechnology (ICN2), Bellaterra, Spain; 4 National Graphene Institute, and Faculty of Biology, Medicine & Health, University of Manchester, Manchester, UK; 5 Comparative Medicine, Karolinska Institutet, Stockholm, Sweden

**Keywords:** Nanoscience and technology, Microbiology, Nanobiotechnology

## Abstract

The gut microbiome produces metabolites that interact with the aryl hydrocarbon receptor (AhR), a key regulator of immune homoeostasis in the gut^1,2^. Here we show that oral exposure to graphene oxide (GO) modulates the composition of the gut microbiome in adult zebrafish, with significant differences in wild-type versus *ahr2*-deficient animals. Furthermore, GO was found to elicit AhR-dependent induction of *cyp1a* and homing of *lck*^+^ cells to the gut in germ-free zebrafish larvae when combined with the short-chain fatty acid butyrate. To obtain further insights into the immune responses to GO, we used single-cell RNA sequencing to profile cells from whole germ-free embryos as well as cells enriched for *lck*. These studies provided evidence for the existence of innate lymphoid cell (ILC)-like cells^3^ in germ-free zebrafish. Moreover, GO endowed with a ‘corona’ of microbial butyrate triggered the induction of ILC2-like cells with attributes of regulatory cells. Taken together, this study shows that a nanomaterial can influence the crosstalk between the microbiome and immune system in an AhR-dependent manner.

## Abstract (web summary)

Although the toxicity of graphene‐based nanomaterials on human health has been extensively studied, their impact on the microbiome remains poorly understood. Using zebrafish as a model, we show that graphene oxide modulates the immune system in a microbiome‐dependent manner through a mechanism mediated by the aryl hydrocarbon receptor. The study suggests an interplay among graphene‐based nanomaterials, microbiome and innate immune system.

## Main

The increasing exploitation of nanomaterials including graphene-based materials necessitates a comprehensive evaluation of the potential effects of these materials on human health^4^. However, although the interactions of nanomaterials with the immune system have been addressed, their impact on the microbiome of the host remains to be understood. Furthermore, studies are needed to address whether graphene-based materials or other nanomaterials modulate immune responses via effects on the microbiota and/or its metabolites. The microbiome, our ‘forgotten organ’, is involved in the regulation of multiple signalling pathways in the host, and there exists bidirectional communication between the gut microbiome and the immune system^5^. The gut microbiome produces numerous metabolites including short-chain fatty acids (SCFAs) such as acetate (AA), butyrate (BA) and propionate (PA)^5^. SCFAs, in turn, signal to host cells in the gut and beyond through several distinct mechanisms^2^. Recent work has shown that BA regulates the aryl hydrocarbon receptor (AhR) and its target genes (including genes encoding members of the cytochrome P450 family, such as *CYP1A1*) in the liver and intestine^6^, whereas other studies have implicated BA in the modulation of AhR expression or the expression of other AhR ligands^7,8^. AhR controls intestinal epithelial cell regeneration, mediates anti-inflammatory responses and modulates type 3 innate lymphoid cell (ILC3) polarization^2^. The activation of AhR also facilitates the induction of tolerogenic regulatory T cells (T_regs_), and a recent study has shown that the AhR pathway regulates the ILC2–ILC3 balance in the gastrointestinal (GI) tract to ensure an appropriate immune response against pathogens^9^. Thus, alterations of the gut microbiota could lead to changes in the pool of AhR ligands, subsequently affecting gut immunity in the host.

**Fig. 1:**
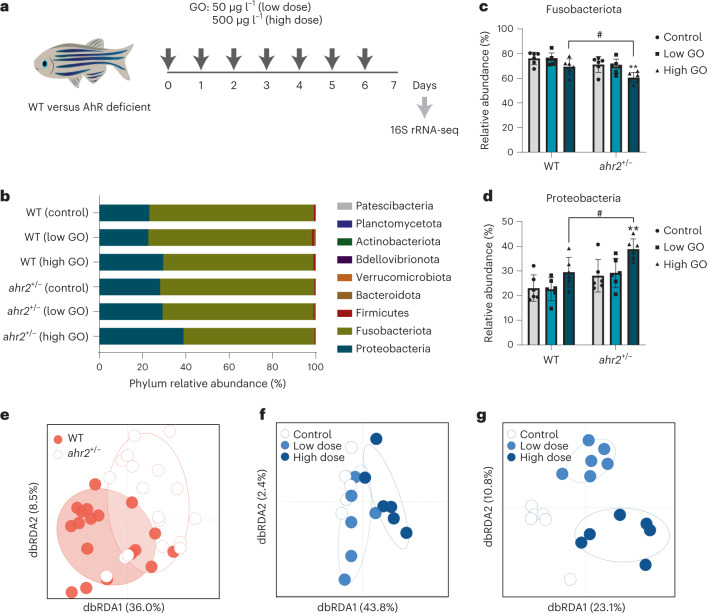
AhR-dependent changes in the gut microbiome of adult zebrafish. **a**, Experimental design for the seven-day exposure regimen in adult zebrafish (WT and *ahr2*^+/−^). **b**, The most abundant bacteria phyla of the gut microbiota among genotypes and treatments. Each bar represents the average of six individuals in each condition. **c**,**d**, Relative phylum abundance of Fusobacteriota (**c**) and Proteobacteria (**d**) in WT versus *ahr2*^+/−^ fish exposed to GO. The error bars represent the mean values ± s.d. of six individuals. Significant differences between the treatments and genotypes are shown. Two-way analysis of variance using Tukey’s multiple comparisons test was used to analyse the statistical differences (Fusobacteriota, **p = 0.0065, #*p* = 0.0265; Proteobacteria, **p = 0.0055, #*p* = 0.0186). **e**, Supervised analyses of the microbiota composition between the two genotypes. **f**,**g**, Impact of GO on gut microbiota composition among WT (**f**) and *ahr2*^+/−^ (**g**) zebrafish. dbRDA, distance-based redundancy analysis. Differential abundances of ASVs are shown in Supplementary Fig. 5. Credit: fish in **a**, Adobe Stock. Source data

In this study, we first determined whether graphene oxide (GO) can modulate the gut microbiota composition, and whether the AhR plays a role in shaping the microbiota in unexposed or exposed animals. To this end, we used the zebrafish (*Danio rerio*) as a model^10^. We previously reported on the structural properties of GO in E3 medium^11^ (Supplementary Fig. 1 shows the corresponding results in other relevant media). GO was determined to be endotoxin free before exposing the animals (Supplementary Fig. 2). We exposed wild-type (WT) and AhR-deficient zebrafish to GO (50 or 500 µg l^–1^) continuously for seven days at which time intestines were dissected and samples were harvested for 16S rRNA gene sequencing (Fig. 1a). A transmission electron microscopy (TEM) analysis of the dissected intestines showed that GO was present in the gut lumen admixed with bacteria (Supplementary Fig. 3). High-magnification images revealed GO sheets in close apposition with microvilli (low dose) and signs of cellular uptake of GO (high dose). Epithelial cells at mucosal barriers represent the very first line of immune defense^12^, and the hyperplasia of goblet cells—specialized mucin-secreting epithelial cells—is a hallmark of type 2 immunity^13^. We determined the expression of goblet cells by using the Alcian blue and periodic acid–Schiff reagent and observed a significant increase in goblet cells in animals exposed to GO (50 µg l^–1^) (Supplementary Fig. 4). Furthermore, 16S rRNA gene sequencing revealed that the phyla Proteobacteria and Fusobacteria dominated the intestinal microflora in both WT and AhR-deficient (*ahr2*^+/−^) zebrafish (Fig. 1b). Exposure to GO shifted the relative abundance from Fusobacteria to Proteobacteria, and this effect was more pronounced in AhR-deficient animals (Fig. 1c,d). Interestingly, 16S rRNA gene sequencing revealed a significant shift in the ratio of Firmicutes to Bacteroidetes following oral exposure of mice to GO (2.5 mg kg^−1^ per day for seven days)^14^. The present data showed that the gut microbiota composition was significantly different between the two genotypes (*R*^2^, 21%; *p* = 0.0004) and between the exposure groups (*R*^2^, 26%, *p* = 0.0001) (Fig. 1e and Supplementary Tables 1 and 2). GO exposure explained 46% of the variation in microbiota composition in WT animals (Fig. 1f), whereas in AhR-deficient animals, GO exposure explained 34% of the variation (Fig. 1g). Differential abundance testing of the amplicon sequence variants (ASVs) of the bacterial 16S rRNA gene sequences in WT and AhR-deficient animals exposed to GO are summarized in Supplementary Fig. 5. Notably, in WT animals, high-dose exposure resulted in an enrichment of *Vibrio*, *Pseudomonas* and *Aeromonas*, consistent with previous studies of known AhR agonists^15,16^, whereas in AhR-deficient animals, similar effects were noted in both low-dose and high-dose groups. However, the relative abundance of individual members of the gut microbiota does not necessarily correlate with their immunomodulatory effects^17^. No differences in ASV abundance were noted between males and females (Supplementary Table 1). In contrast, another work^18^ found that chronic exposure (25 days) to a high dose (5 mg l^–1^) of GO elicited differences between female and male zebrafish at the phylum and genus levels. In sum, week-long exposure to GO substantially affected the gut microbiota composition in adult zebrafish, which, in turn, was modulated by the AhR.

**Fig. 2:**
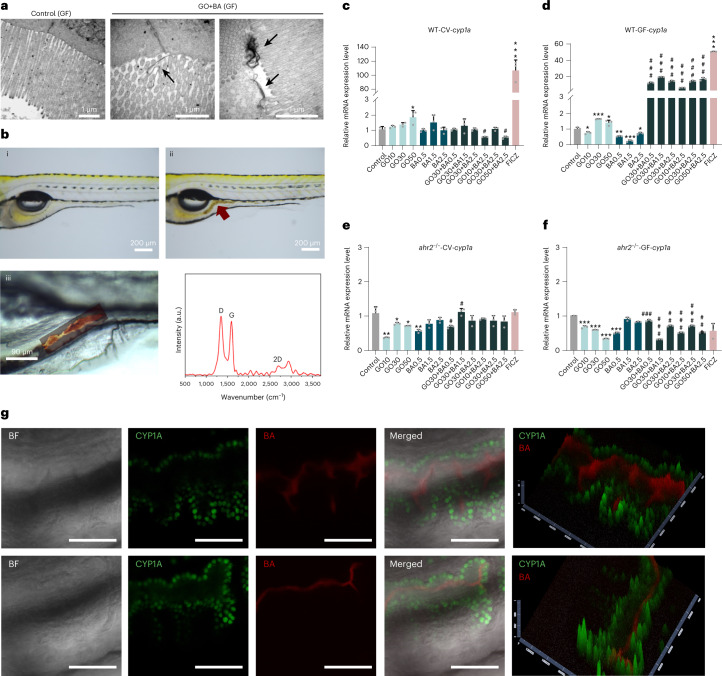
GO plus BA trigger AhR-dependent CYP induction in GF zebrafish. **a**, GO visualized by TEM analysis. The arrows indicate GO sheets interacting with microvilli in the gut of GF zebrafish larvae exposed to 5 µg ml^–1^ of GO for 24 h. Scale bars, 1 μm. **b**, Light microscopy ((i) and (ii)) and Raman confocal mapping (iii) to verify the presence of GO in the gut. The analysis was done on 5 dpf zebrafish exposed to 5 µg ml^–1^ of GO for 24 h. The spectra shown represent the average of 10,000 spectra across the whole area scan. **c**,**d**, Relative mRNA expression of *cyp1a* in WT-CV (**c**) and WT-GF (**d**) larvae. **e**,**f**, Relative mRNA expression of *cyp1a* in *ahr2*^−/−^ CV (**e**) and *ahr2*^−/−^ GF (**f**) larvae. FICZ was used as a positive control. Data are presented as mean values ± s.d. of three independent experiments (*n* = 3). Student’s *t*-test (two sided) was used for the analysis of comparisons between control and the indicated treatments (*p < 0.05, **p < 0.01, ***p < 0.001), and for comparisons between BA versus GO+BA (#*p* < 0.05, ##*p* < 0.01, ###*p* < 0.001). **g**, Visualization of *cyp1a* induction using Tg(*cyp1a*:GFP) zebrafish larvae under GF conditions following exposure to the combination of GO (30 μg ml^–1^) and resorufin butyrate (5 μM). BA (red) was found in the gut lumen, and *cyp1a* induction (green) was noted in the GI epithelial cells (Supplementary Figs. 8 and 9 show additional positive and negative controls). The upper and lower rows are from two different individuals. The pseudo-three-dimensional images were generated with the 2.5D tool in ZEN 3.0, and the highest-intensity values are represented by the greatest extension in the *z* axis. BF, bright field. Scale bars, 50 μm. Source data

To further investigate the importance of the microbiome, we generated germ-free (GF) zebrafish embryos on a WT and AhR-deficient background (Supplementary Fig. 6). Previous work has demonstrated that bacterial communities from adult zebrafish intestines synthesize all the three main SCFAs^19^, and BA has also been detected in situ (in adult zebrafish)^20^. Importantly, using the human intestinal epithelial cell line HT-29, we found that only BA (not AA or PA) was able to trigger AhR activation (Supplementary Fig. 7). Similarly, others have shown that BA induced *CYP1A1* expression in HT-29 cells^6^. Using TEM, we could also show that GO (30 μg ml^–1^) was internalized by differentiated HT-29 cells following a 24 h exposure, in contrast to a previous study in which differentiated Caco-2 cells were used to model the GI barrier^21^. Thus, GO is taken up by intestinal cells in vitro and in vivo (as shown above) and may therefore serve to ‘deliver’ BA to these cells. The exposure of GF zebrafish larvae (5 days post fertilization (dpf)) to GO (5 µg ml^–1^) for 24 h resulted in the presence of GO in the GI tract, as evidenced by TEM (Fig. 2a) and Raman spectroscopy/microscopy (Fig. 2b). The TEM analysis showed that GO was located on the surface of the epithelial cell microvilli. GO exposure did not adversely affect the overall architecture of the GI tract, although a certain degree of microvilli and epithelial cell membrane damage was noted. We then monitored the induction of *cyp1a* as a marker of AhR activation using reverse transcription quantitative polymerase chain reaction (RT-qPCR). To this end, embryos were exposed to GO (10, 30 and 50 µg ml^–1^), BA (0.5, 1.5 and 2.5 mM) or to a combination of GO and BA (GO+BA) at the indicated concentrations for 24 h. We used the high-affinity AhR ligand 6-formylindolo[3,2-*b*]carbazole (FICZ)^22^ as a positive control. FICZ triggered a marked induction of *cyp1a* in conventional (CV) and GF embryos, and this was nullified in *ahr2*^−/−^ embryos (Fig. 2c–f). GO and BA, alone or in combination, had no or modest effects in CV embryos. In contrast, in GF embryos, the combined exposure to GO+BA triggered a significant (~20-fold) induction of *cyp1a*, and this was not seen in *ahr2*^−/−^ embryos (Fig. 2d,f), confirming the role of AhR. We also utilized transgenic Tg(*cyp1a*:GFP) zebrafish derived under GF conditions and could show that GO+BA elicited *cyp1a* induction in the GI epithelium (Fig. 2g), whereas red fluorescent BA was detected in the gut lumen. FICZ also prompted *cyp1a* induction in the liver and gut, whereas the response to BA alone or GO alone was less pronounced (Supplementary Figs. 8 and 9).

**Fig. 3:**
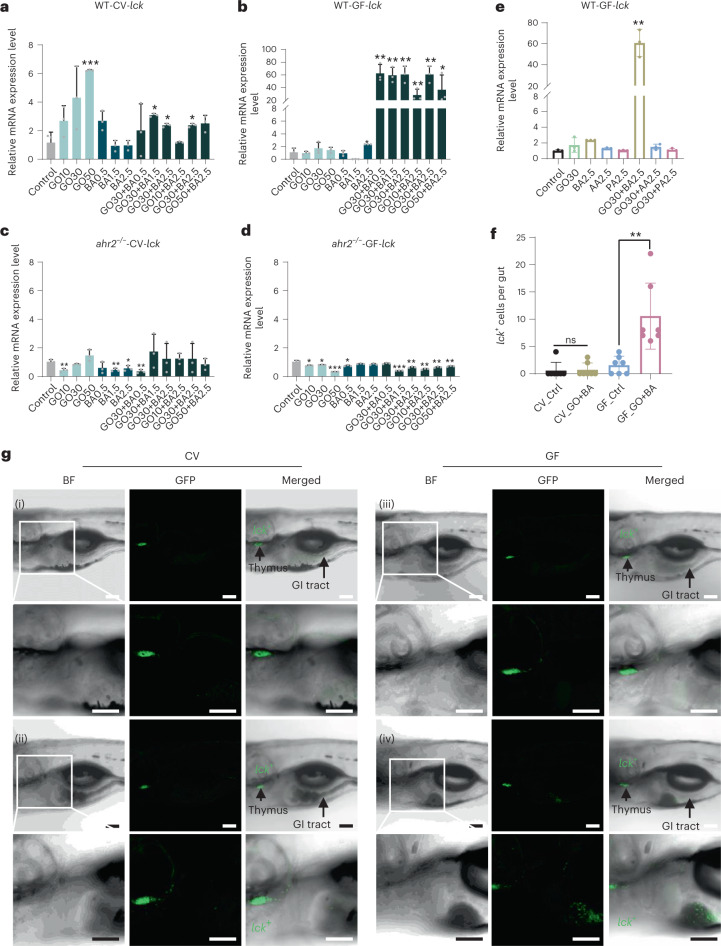
GO plus BA trigger AhR-dependent homing of *lck*^+^ cells in GF fish. **a**–**d**, Relative mRNA expression of *lck* in WT-CV (**a**), WT-GF (**b**), *ahr2*^−/−^-CV (**c**) and *ahr2*^−/−^-GF (**d**) zebrafish larvae on exposure to GO alone, BA alone or GO+BA at the indicated concentrations. Supplementary Fig. 10 shows the additional gene profiling results. Data are presented as mean values ± s.d. of three independent experiments (*n* = 3). Student’s *t*-test (two sided) was used for the analysis of comparisons between control and the indicated treatments (*p < 0.05, **p < 0.01, ***p < 0.001). **e**, PCR analysis of *lck* following the exposure to GO and/or various SCFAs in WT-GF zebrafish larvae. AA, acetic acid; BA, butyric acid; PA, propionic acid. Student’s *t*-test (two sided) was used for the comparison between control and exposed larvae (**p = 0.0014). **f**, Quantification of *lck*^+^ cells homing to the gut. A significant increase in *lck*^+^ cells in the gut was observed on GO+BA exposure under GF conditions, but not in CV zebrafish. Student’s *t*-test (two sided) was used for the analysis of comparisons between control and the treatments (ns = no significant difference; **p = 0.0055). The numbers of *lck*^+^ cells were quantified based on seven individuals per group. **g**, Visualization of *lck*^+^ cells using Tg(*lck*:GFP) zebrafish larvae under CV and GF conditions exposed as follows: (i) CV fish (control), (ii) CV fish (GO+BA), (iii) GF fish (control), (iv) GF fish (GO+BA) (Supplementary Fig. 11 shows the experiments with resorufin butyrate). Scale bars, 100 μm. Source data

Next, we asked whether GO alone or in combination with the microbial metabolite BA would elicit immune responses in zebrafish embryos (Fig. 3a–d). Indeed, we observed a profound induction (~60-fold) of *lck* (lymphocyte-specific protein tyrosine kinase) in GF embryos exposed to GO+BA, whereas neither GO or BA alone had any effect. This was not the case in CV embryos nor did we detect the induction of *lck* in AhR-deficient zebrafish. Furthermore, neither AA nor PA (alone or in combination with GO) upregulated *lck* in GF zebrafish (Fig. 3e). Since we applied embryos at 5 dpf, corresponding to the early larval stage, the immune responses may be mainly attributed to the innate immune system^23^. Hence, GO+BA triggered the induction of *lck*, a molecular marker that is shared by all the three innate lymphoid cell (ILC) subtypes^24^, in a strictly AhR-dependent manner, and this was observed only in GF zebrafish. Furthermore, gene expression profiling showed that the transcription factor genes, namely, *gata3* and *stat6*, and the cytokine-encoding genes, namely, *il4* and *il13*, corresponding to ILC2 cells were upregulated in the GF embryos (Supplementary Fig. 10), whereas genes related to ILC1 (*tbet*, *ifn*-*γ*) and ILC3 (*rorca*, *il22*) cells were not upregulated. To confirm the induction of *lck* in situ, transgenic Tg(*lck*:GFP) zebrafish larvae raised under CV and GF conditions were exposed to GO+BA. We found that GO+BA prompted the homing of *lck*^+^ cells to the gut (Fig. 3f). This was only observed in GF zebrafish (Fig. 3g shows the representative images and Supplementary Fig. 11 shows the visualization of red fluorescent BA in the gut and *lck* (green) in the thymus and GI tract).

**Fig. 4:**
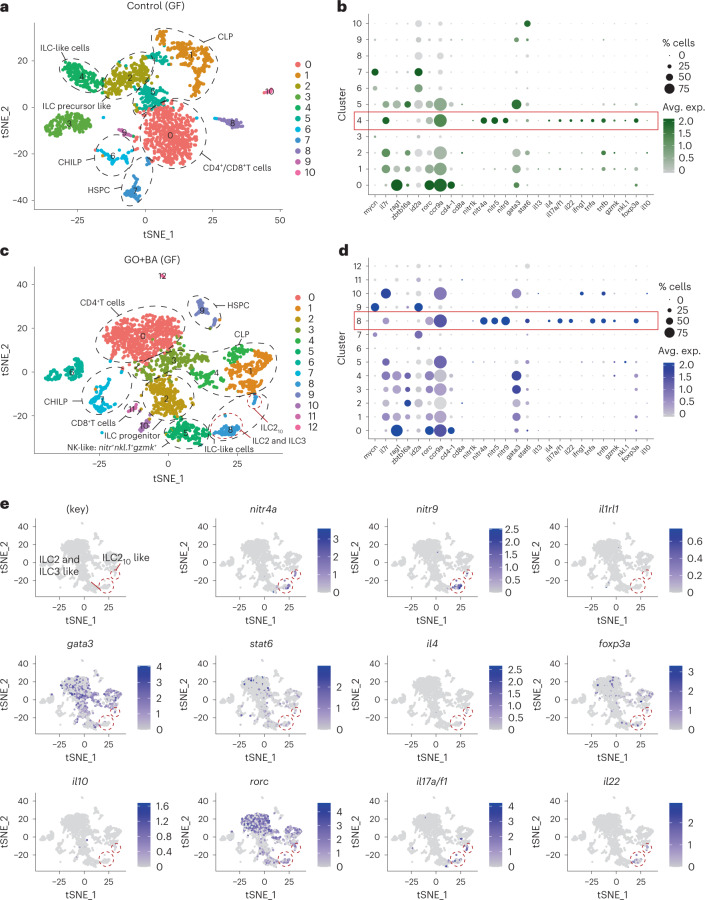
scRNA-seq analysis of *lck*^+^-enriched cells collected from GF zebrafish. **a**,**c**, Two-dimensional projection of tSNE analysis of 10x RNA-seq data showing the heterogeneity of *lck*^+^ cells in controls (**a**) and GO+BA-exposed embryos (**c**). **b**,**d**, Dot plots show the average expression level of target genes in each of the clusters in control (**b**) and GO+BA-exposed larvae (**d**). The size of the dots indicates the percentage of cells within the cluster that express the gene in question. The red boxes delineate cluster 4 (corresponding to ILC-like cells) in control (**b**) and cluster 8 (corresponding to ILC-like cells) in GO+BA-exposed fish (**d**). **e**, Feature plots of the ILC-like cluster in GO+BA-exposed larvae (cluster 8 in **c** and **d**), which, in turn, is shown to comprise ILC2-like cells (*nitr*^+^*gata3*^+^*il4*^+^*il13*^+^) and ILC3-like cells (*nitr*^+^*rorc*^+^*il17a*/*f1*^+^*il22*^+^), as well as ILC2 cells with attributes of regulatory ILC-like cells (ILC2_10_ cells) (*nitr*^+^*gata3*^+^*foxp3a*^+^*il10*^+^). Supplementary Fig. 17 provides additional information and Supplementary Fig. 16 shows the feature plots of ILC-like cell markers in the control sample (corresponding to cluster 4 in **a** and **b**).

**Extended Data Fig. 1:**
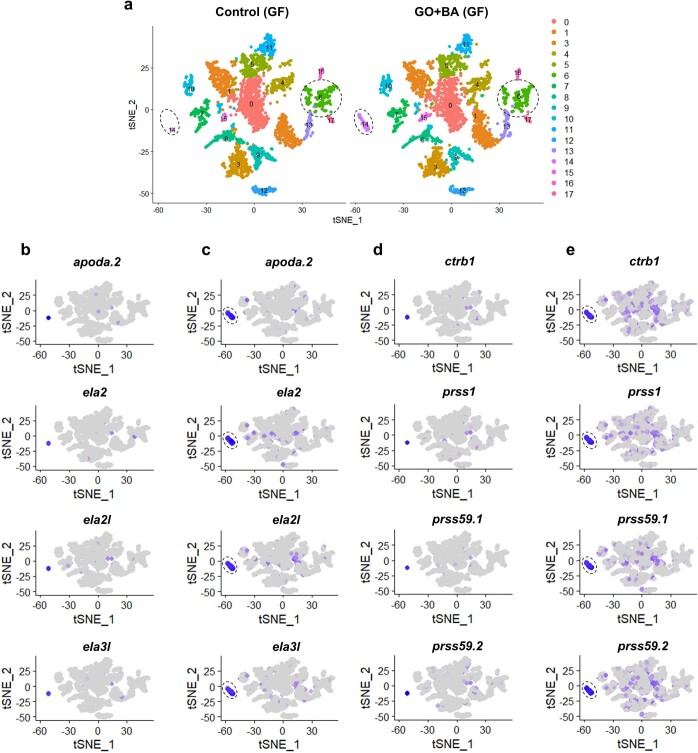
Integrated analysis of wild-type (WT) germ-free (GF) control versus GO+BA samples. (**a**) 2D projection of the tSNE analysis showing *lck*^*+*^ lymphocytes (cluster 6) and the cluster corresponding to the liver and pancreas (cluster 14) which is noticeably expanded in the GO+ BA fish. Below are the feature plots showing the expression of genes involved in lipid metabolism (*apoda.2*) and proteolysis (*ela3l, ela2l, ela2, ctrb1, prss1, prss59.1, prss59.2*), respectively, in the GF control samples (**b**,**d**) versus GO+BA samples (**c**,**e**).

Importantly, a recent single-cell transcriptional analysis revealed the presence of ILC-like cells in the gut of adult zebrafish^25^. However, ascribing specific cellular phenotypes on the basis of bulk analysis of gene expression by qPCR is challenging. Therefore, to identify ILC-like cells in zebrafish embryos and to determine the impact, if any, of GO+BA on these cells, we performed single-cell RNA sequencing (scRNA-seq) on cells from whole embryos as well as on cells enriched for *lck* (Supplementary Fig. 12a,b). To this end, cells from WT embryos and *lck*^+^ cells enriched from Tg(*lck*:GFP) embryos raised under GF conditions and exposed to GO+BA or not were collected (Supplementary Fig. 12c,d) and subjected to scRNA-seq using the 10x Genomics technology, as detailed in Methods (Supplementary Figs. 13 and 14 show the quality control (QC) of these transcriptomics data). The transcriptomics analysis of whole zebrafish embryos allowed us to identify an *lck*^+^ cell population in the control samples with markers of both T cells and ILCs, whereas in GO+BA-exposed zebrafish, two separate *lck*^+^ cell populations were identified, out of which one corresponded to T cells and the other to ILC-like cells (Supplementary Fig. 15). Furthermore, the integrated analysis of both samples revealed the expansion of a cell population expressing markers of pancreas and liver in the GO+BA-exposed larvae (Extended Data Fig. 1). This may imply that the presence of GO with a ‘corona’ of SCFAs in the gut is sensed as ‘food’, leading to the induction of genes encoding digestive enzymes (for example, serine proteases) and genes involved in lipid metabolism (Extended Data Fig. 1). We also noted that *cyp1a* was induced in the cluster identified as intestinal cells in GF embryos exposed to GO+BA, in line with the results obtained in Tg(*cyp1a*:GFP) zebrafish. However, ILCs comprise only a small fraction of lymphocytes present at mucosal barriers^26^. To refine our approach, we, therefore, performed scRNA-seq on *lck*^+^ cells sorted from GF Tg(*lck*:GFP) larvae. Our analysis showed that a distinct cell population (cluster) displaying markers of ILCs was present in the control (Fig. 4a), in line with a previous study in which ILC-like cells were identified in dissected intestines of adult zebrafish^25^. The relevant genes are shown in Fig. 4b, and the red box delineates cluster 4 (corresponding to ILC-like cells). Furthermore, on exposure to GO+BA, a cluster corresponding to ILC-like cells could be identified (Fig. 4c) which, in turn, was shown to comprise ILC2-like cells (*nitr*^+^*gata3*^+^*il4*^+^*il13*^+^) and ILC3-like cells (*nitr*^+^*rorc*^+^*il17a*/*f1*^+^*il22*^+^) as well as ILC2 cells with attributes of regulatory ILC-like cells known as ILC2_10_ cells^27^ (*nitr*^+^*gata3*^+^*foxp3a*^+^*il10*^+^) (Fig. 4d). Feature plots of the ILC-like cluster in GO+BA-exposed larvae (Fig. 4c,d, cluster 8) are displayed in Fig. 4e. The corresponding feature plots for the control sample are shown in Supplementary Fig. 16 (note that the gene encoding IL-10 is not present in this cluster). Furthermore, the specific markers in the cell population corresponding to ILC2_10_-like cells in exposed larvae are shown in Supplementary Fig. 17. It is noted that although it has been hypothesized that a regulatory ILC population exists^28^, such cells (that is, an ILC subset expressing FOXP3) have thus far not been identified in mice or humans^29^. However, IL-10-producing ILC2 cells have been associated with regulatory activities^29^. It is, thus, relevant to note that we identified a subset of cells in GF zebrafish with markers of ILC2 cells along with *il10* and *foxp3*. These cells were also found to express *il1rl1* (also known as *st2*), encoding a receptor for IL-33 (Supplementary Fig. 17). Previous studies in mice have shown that IL-10-producing ILC2 cells can be generated following the activation by alarmins such as IL-33 and retinoic acid^27^. However, the gene encoding IL-33 is absent from the zebrafish genome, and one may speculate that another ‘IL-33-like’ factor may be involved. Thus, our studies provided evidence for the existence of ILC-like cells in zebrafish embryos and suggested plasticity within ILC lineages in GF zebrafish exposed to GO+BA. Further studies are required to functionally characterize these cells.

Previous studies in mice have shown that the AhR plays a role in the maintenance and function of ILC3s in the gut^2^. However, GF conditions did not affect the development of the latter cells (reviewed in another work^2^). Here we uncovered a novel aspect of gut immunity where a nanomaterial (GO) in combination with a microbial metabolite (BA) was shown to elicit AhR-dependent type 2 immune responses in GF zebrafish with the induction of ILC2-like cells displaying attributes of regulatory cells. In conclusion, this study demonstrated that GO influences the crosstalk between the gut microbiome and immune system with the induction of a type 2 immune response. Type 2 immunity is best known for its protective role against helminth infections, as well as for its pathogenic role in allergic diseases such as asthma^30^. Our findings imply that the immune system ‘senses’ GO+BA as a pathogen. This has important implications for our understanding of the hazard potential of graphene-based materials and other nanomaterials and places AhR at the nexus of the bidirectional communication between the gut microbiome and innate immune system.

## Methods

### Characterization of GO

GO prepared by Hummers’ method was obtained from Graphenea (Spain). The full physicochemical characterization of the GO suspensions in H_2_O and E3 medium has been previously reported^11^. Here we provide information on GO suspensions in H_2_O and different cell culture media, that is, Dulbecco’s modified Eagle medium (DMEM) (used for the HT-29 cell line) and Roswell Park Memorial Institute (RPMI) (used for primary macrophages). TEM and atomic force microscopy was performed as previously described^11^. Briefly, carbon-film-coated grids were pretreated with a glow discharge using a current of −25 mA and for 30 min duration. Ten microlitres of the solutions at 50 µg ml^–1^ were deposited on the grid, removing excess sample after 2 min of deposition time. TEM images were acquired at 80 kV, and the size distribution analysis was determined using ImageJ software version 1.5. Atomic force microscopy images were acquired using a Bruker Multimode 8 atomic force microscope in the tapping mode with an OTESPA probe.

### Endotoxin detection

GO suspensions were evaluated for endotoxin content by using the TNF-α expression test based on primary human monocyte-derived macrophages, as previously described^31^. The cells were isolated from buffy coats obtained from the Karolinska University Hospital. The samples are completely anonymized, and the data cannot be traced back to the individual donors. Human monocyte-derived macrophages were exposed to GO (5–75 μg ml^–1^) for 24 h and cell viability was evaluated using the alamarBlue assay (ThermoFisher Scientific)^32^. Human monocyte-derived macrophages were then incubated for 24 h with GO at 25 μg ml^–1^ in the presence or absence of the lipopolysaccharide inhibitor polymyxin B (10 μM). Lipopolysaccharide (0.01 µg ml^–1^) was used as a positive control. Cell culture supernatants were collected and TNF-α levels were determined by enzyme-linked immunosorbent assay (MabTech). A standard curve was established based on lipopolysaccharide-induced TNF-α.

### AhR reporter cells

HT-29-Lucia AhR reporter cells established from the human HT-29 colon adenocarcinoma cell line were obtained from InVivoGen. The cells were initially cultured in DMEM supplemented with 4.5 g l^–1^ glucose, 2 mM l-glutamine, 10% foetal bovine serum (FBS), 100 U ml^–1^ penicillin, 100 μg ml^–1^ streptomycin and 100 μg ml^–1^ Normocin. Following at least two passages, the growth medium was supplemented with Zeocin (100 µg ml^–1^). For the AhR activity assay, cells were detached with trypsin, centrifuged and resuspended in the test medium without Normocin and Zeocin at a density of 2.8 × 10^5^ cells ml^–1^. The cells were exposed to AA, BA or PA (Sigma-Aldrich); FICZ (Sigma-Aldrich) (200 nM) was used as a positive control. Then, cell supernatants were transferred into a 96-well plate, and QUANTI-Luc assay solution was added. The measurements were recorded using a Tecan Infinite F200 plate reader.

### HT-29 cell differentiation

HT-29 cells were cultured in RPMI 1640 supplemented with 10% FBS, 2 mM l-glutamine, 100 U ml^–1^ penicillin, 100 μg ml^–1^ streptomycin and 2 g l^–1^ glucose for 21 days, as described^33^. The cell medium was changed every other day until the cells reached confluence. Thereafter, the medium was changed every day for 21 days. The differentiated cells were then exposed to GO (30 μg ml^–1^) for 24 h. Then, the cells were washed, harvested using trypsin–EDTA (0.25%) and fixed in 2.50% glutaraldehyde in 0.1 M phosphate buffer (pH 7.4). The samples were then processed for TEM analysis, as described in the ‘Ultrastructural analysis’ section.

### Zebrafish genotyping

Zebrafish were housed and experiments were conducted in compliance with national ethical guidelines, and the present study was approved by the regional committee for animal experiments in Stockholm (ethical permit no. 14049-2019). Zebrafish embryos carrying a point mutation in *ahr2* (*ahr2*^hu3335^) were generated at the Wellcome Sanger Institute and provided by the European Zebrafish Resource Center at Karlsruhe Institute of Technology. Offspring were raised to adulthood and genotyped for the *ahr2*^hu3335^ point mutation with DNA isolated from fin clips^34^. DNA was extracted using the QIAamp DNA Mini Kit (QIAGEN) and amplified with the point mutation detection primers (a*hr2*-mut-F, TATTGCTAGGCAGAGAGCAC; *ahr2*-mut-R, GATGTCTTCTGTGATGATTTCG) using the DreamTaq Green PCR Master Mix (ThermoFisher). The PCR product was purified with ExoSAP-IT Express reagent (Applied Biosystems), and loaded on the ABI 3730 PRISM DNA analyser (Applied Biosystems) for DNA sequencing. Zebrafish determined to be WT (a*hr2*^+/+^), heterozygous (*ahr2*^+/−^) or homozygous (*ahr2*^−/−^) for the point mutation in *ahr2* were used for further experiments (adult zebrafish, 4.5 months old; larvae, 5 dpf).

### Adult zebrafish exposure

Adult zebrafish of different genotypes (WT, *ahr2*^+/−^ and *ahr2*^−/−^) were continuously exposed to GO (50 µg l^–1^ or 500 µg l^–1^) for seven days. For WT, six female and six male fish were included, whereas for the other genotypes, three female and three male fish were included. The zebrafish were housed together before the genotyping (see above) and were then housed in separate fish tanks for one week before the exposures to GO. During the seven-day exposure, the fish were fed once per day in the morning with an approximately equal amount for each tank. The fish water was refreshed 1 h after the feeding and GO was added to the exposure groups. At day 7, the fish were sacrificed with tricaine, and the intestines were dissected under a stereomicroscope and fixed in 2.5% glutaraldehyde in 0.1 M phosphate buffer at pH 7.4 and stored at 4 °C for TEM analysis (WT), or in 4% formaldehyde for histopathological examination (WT) or stored at −80 °C for subsequent 16S rRNA gene sequencing (WT, *ahr2*^+/−^). The *ahr2*^+/−^ zebrafish were used for the analysis of the gut microbiome composition as the survival of some *ahr2*^−/−^ fish in the high-dose GO exposure group was compromised.

### Histopathology

After fixation in 4% formaldehyde for at least 24 h, the intestines were dehydrated in ethanol and embedded in paraffin using an embedding station (Tissue-Tek, Sakura Finetek). The paraffin-embedded tissues were then cut using a microtome (Microm HM 360, Marshall Scientific). The thickness of each slice was 5 µm. The slices were deparaffinized following the steps of xylene for 5 min, xylene for 5 min, 100% ethanol for 3 min, 95% ethanol for 3 min and distilled water for 3 min. Sections were stained with haematoxylin and eosin for general morphological examination, and Alcian blue and periodic acid–Schiff reagent (ThermoScientific) for goblet cell identification using a Zeiss Axioplan microscope equipped with an Olympus SC30 digital camera. The numbers of goblet cells per villus are presented as average results of six slices per condition.

### Ultrastructural analysis

TEM analysis^35^ of the GI tract of control and exposed animals was performed on 5 dpf larvae and dissected intestines of adult zebrafish. Following the primary fixation, samples were rinsed with 0.1 M phosphate buffer followed by post-fixation in 2% osmium tetroxide in 0.1 M phosphate buffer at pH 7.4 at 4 °C for 2 h. The samples were then ethanol dehydrated stepwise followed by stepwise acetone/LX-112 infiltration and finally embedded in LX-112. Semi- and ultrathin sections were prepared on a Leica EM UC7 ultramicrotome. The ultrathin sections were then contrasted with uranyl acetate followed by Reynolds lead citrate and examined using a Hitachi HT7700 transmission electron microscope operating at 100 kV. Digital images were acquired using a 2k × 2k Veleta charge-coupled device camera.

### 16S rRNA gene sequencing

Total DNA of the zebrafish gut samples was extracted using the QIAamp DNA Mini Kit (QIAGEN). The amplicon PCR was then performed with bacterial universal primers to target the V3 and V4 regions of the 16S rRNA gene using the forward primer TCGTCGGCAGCGTCAGATGTGTATAAGAGACAGCCTACGGGNGGCWGCA and the reverse primer GTCTCGTGGGCTCGGAGATGTGTATAAGAGACAGGACTA CHVGGGTATCTAATCC. The KAPA HiFi HotStart ReadyMix (KAPA Biosystems) was used for the PCR reactions. Index PCR was performed with the Nextera XT Index Kit (Illumina), followed by cleanup with AMPure XP beads. The concentrations of the DNA libraries were quantified by an Agilent 2100 Bioanalyser (Agilent Technologies). DNA libraries were then sequenced on the Illumina MiSeq system to generate raw paired-end reads (2 × 150 bp).

### Gene sequencing analysis

#### Preprocessing

Primers were removed from sequences using cutadapt (version 2.9) and sequences were quality checked by FastQC (version 0.11.9) and combined using multiqc (version 1.9.dev0). ASVs were inferred from the bacterial 16S rRNA gene sequences were using the DADA2 package^36^ (version 1.14.1). Forward and reverse reads were truncated after 282 and 222 bp, respectively, and further filtered using the function filterAndTrim with default options, with the exception that the maximum expected error rate was set at 2. The rest of the functions in the DADA2 pipeline were performed with default options, with the exception that before sample inference, that is, removing sequencing errors, all the samples were pooled rather than handled sample wise (default). The ASVs of 16S rRNA gene sequences were assigned to taxa using the SILVA taxonomic training data formatted for DADA2 (version 138)^37^. Before the normalization step, non-bacterial sequences were removed. ASVs were then normalized using cumulative sum scaling^38^. The steps in the analysis of the data from preprocessing to further downstream analyses were done in the R environment (3.6.2, R Core Team, 2019).

#### Statistical analysis

Before unsupervised and supervised analyses of the preprocessed and normalized data, ASVs occurring only in one sample were removed. To investigate the overall variation in the gut microbiota of WT and AhR-deficient zebrafish, principal coordinate analysis was performed using the function cmdscale in the R package vegan^39^. Supervised analyses were, in turn, used to study the effect of GO exposure on the gut microbiota of WT and *ahr2*^+/−^ animals, taking into account the gender of the animals. Permutational multivariate analysis of variance (PERMANOVA) and distance-based redundancy analysis (dbRDA) were performed using the functions adonis and dbrda, respectively, in the R package vegan. In the PERMANOVA and dbRDA analyses, exposure, gender and genotype were applied as categorical variables to supervise the microbiota composition. Between-sample Bray–Curtis distances were used in principal coordinate analysis, PERMANOVA and dbRDA. Statistical significances were based on 9,999 random permutations. To determine which ASVs were differently abundant in exposures and genotypes, the function fitFeatureModel was used in R package metagenomeSeq (retrieved from https://cbcb.umd.edu/software/metagenomeSeq).

### GF zebrafish derivation

The generation of GF zebrafish followed previously established protocols^40^. In brief, 2 hpf embryos were transferred to Petri dishes with sterile E3 medium, supplemented with ampicillin (100 μg ml^–1^), kanamycin (5 μg ml^–1^) and amphotericin B (250 ng ml^–1^), and incubated at 28 °C. At 50% epiboly up to the shield stage (6 hpf), the embryos were surface disinfected with 0.1% polyvinylpyrrolidone-iodine for exactly 2 min, followed by 0.003% sterile bleach immersion for 18 min. The embryos were then rinsed with sterile E3 medium, transferred to flasks and incubated at 28 °C. The viability was monitored, and the sterile medium was refreshed daily. At day 4, the hatched embryos were used for sterility validation. Gnotobiotic zebrafish were validated through two different approaches: bacterial growth on Luria broth plates and DNA amplification by bacterial universal primers. To this end, ten embryos were randomly selected from the culture flasks. For the first approach, the embryos were washed and homogenized with 200 μl sterile medium. Then, 100 μl homogenate was spread on the Luria broth plate and incubated at 37 °C overnight. The bacterial colony formation was checked on the next day. For the second approach, DNA of the collected embryos was extracted using the QIAamp DNA Mini Kit (QIAGEN). DNA was amplified with bacterial universal primers (515F/806R) using the DreamTaq Green PCR Master Mix (ThermoFisher). The PCR product was resolved by electrophoresis, and Midori Green Direct (NIPPON Genetics Europe) was used for the visualization of the DNA. The gel image was captured using a Gel Doc EZ System (Bio-Rad).

### Zebrafish larvae exposure

WT and *ahr2*^−/−^ adult zebrafish were maintained at 28.0 ± 0.5 °C on a 14 h:10 h light/dark cycle in the fish breeding circulatory system at the zebrafish core facility at Karolinska Institutet. Two pairs of male/female fish were placed in a single mating tank with a divider one day before spawning. Spawning was triggered by removing the divider in the morning. Embryos were collected after 2 h, washed and then transferred to the E3 medium in a Petri dish. Healthy and fertilized embryos at the same developmental stages were selected and raised up to 5 dpf. Zebrafish larvae at 5 dpf (CV WT, GF WT, CV *ahr2*^−/−^ and GF *ahr2*^−/−^) were exposed to GO or BA or GO+BA for 24 h. Each treatment was performed in three replicates, and each replicate sample contained ten larvae. FICZ (200 nM) was used as a positive control for *cyp1a* induction. After the exposure, the samples were fixed in 2.5% glutaraldehyde for TEM analysis or stored at −80 °C for RT-qPCR, as detailed above.

### Raman confocal analysis

The presence of GO in the GI tract of zebrafish larvae was monitored by Raman confocal analysis^11^. Briefly, larvae exposed to GO for 24 h were washed, anaesthetized in 0.01% tricaine solution and positioned in 1% low-melt agarose on glass slides. Samples were then dried on a plate heater at 50 °C. Raman analysis was performed using a confocal Raman microscope (WITec alpha300 system) with a laser of 532 nm wavelength set at an integration time of 0.5 s and ×60 magnification. The scan area for each sample was adjusted to 50 × 50 µm^2^. The spectra shown represent the average of 10,000 spectra recorded across the whole area scan. GO could be detected on the basis of its characteristic Raman signature, that is, the D band (1,354 cm^−1^), G band (1,582 cm^−1^) and 2D band (2,690 cm^−1^).

### RT-qPCR analysis

RNA extraction was performed on 5 dpf larvae (WT versus *ahr*^*−/−*^) exposed to GO+BA or not using the RNeasy Mini Kit (QIAGEN). RNA concentration was quantified by a NanoDrop spectrophotometer (ThermoFisher). Total RNA (500 ng) was reverse transcribed using the iScript Advanced cDNA Synthesis Kit (Bio-Rad). The transcription of target genes was quantified using a QuantStudio 5 Real-Time PCR System (Applied Biosystems). The reaction mixtures were formulated using Maxima SYBR Green/ROX qPCR Master Mix (ThermoScientific). Thermal cycling conditions were as follows: 95 °C for 10 min, 40 cycles of three-step amplification of 15 s at 95 °C, 30 s at 60 °C and 30 s at 72 °C. Primers (Supplementary Table 3) were purchased from Sigma-Aldrich. The transcription level of each target gene was normalized to *rpl13*. The relative mRNA expression level was calculated relative to control using the 2^−ΔΔCt^ method.

### Tg(*cyp1a*:GFP) reporter strain

Transgenic Tg(*cyp1a*:GFP) zebrafish^41^ were provided by the China Zebrafish Resource Center. GF zebrafish larvae were generated as described above and exposed at 5 dpf to GO (30 µg ml^–1^), red fluorescent resorufin butyrate (Sigma-Aldrich) (5 µM) and a combination of GO and resorufin butyrate for 24 h. FICZ (Sigma-Aldrich) (200 nM) was used as a positive control. After exposure, the larvae were washed, anaesthetized in 0.01% tricaine and positioned in 1% low-melt agarose for analysis by confocal microscopy (Zeiss LSM880, ZEISS). The bright-field images were acquired with the transmitted light detector (T-PMT). The green and red fluorescence images were captured under 488 and 561 nm to visualize *cyp1a* and butyrate, respectively. The images were analysed in ZEN 3.0 software, blue edition (ZEISS). The 2.5D view tool (ZEN) was used to generate pseudo-three-dimensional images and the highest-intensity values are represented by the greatest extension in the *z* axis.

### Tg(*lck*:GFP) reporter strain

Transgenic Tg(*lck*:GFP) zebrafish^42^ were obtained through the European Zebrafish Resource Center. CV and GF larvae were exposed at 5 dpf to a combination of GO (30 µg ml^–1^) and butyrate (2.5 mM) for 24 h. GO and BA were pre-incubated for 1 h before the exposure. After the exposure, the larvae were washed, anaesthetized and positioned for analysis by confocal microscopy (Zeiss LSM880, ZEISS), as described above. The numbers of *lck*-positive cells homing to the gut were manually quantified based on seven fish per group. Resorufin butyrate (5 µM) was also applied to better visualize the interactions between GO, butyrate and *lck*-positive cells under GF conditions. The *z*-stack analysis was performed with the interval of 1 µm of each slice, and *z* projections were made with Fiji (ImageJ)^43^.

### Zebrafish dissociation and cell sorting

GF WT (AB) zebrafish larvae were exposed at 5 dpf to the combination of GO (30 µg ml^–1^) and BA (2.5 mM) for 24 h. GO and BA were pre-incubated for 1 h before the exposure. Twenty larvae were used as one replicate, and four replicates, that is, eighty larvae in total, were used for each condition. After the exposure, zebrafish larvae were dissociated for single-cell suspensions following the published protocol^44^. Briefly, zebrafish larvae were euthanized with 0.01% tricaine for 5 min, collected in a 1.5 ml tube and washed three times with phosphate-buffered saline. The dissociation was initiated by adding 500 μl of pre-warmed enzyme mix containing 460 μl of 0.25% trypsin–EDTA (Gibco) and 40 μl of collagenase (Sigma-Aldrich) (100 mg ml^–1^), followed by mechanical dissociation using P1000 and then P200 pipette tips on a heat block at 30 °C until tissues were no longer visible (about 10 min). The dissociation was then stopped by adding 800 μl DMEM supplemented with 10% FBS. The cell pellets were collected by centrifugation at 1,000 rpm for 5 min at room temperature, followed by washing with phosphate-buffered saline. The cells were then resuspended in 0.5 ml DMEM + 10% FBS. Four replicates from each condition were pooled together at this step and filtered through a 40 μm nylon mesh, with an additional washing step with DMEM + 10% FBS. The cell suspension was then stained for 10 min at room temperature with the fluorescent DNA dye DRAQ7 (Invitrogen) (3 μM) to allow for the exclusion of non-viable cells by using fluorescence-activated cell sorting (BD FACSAria III, BD Biosciences) operating with FCS Express software version 7.0 (DeNovo Software). DRAQ7^–^ cells of each sample were sorted into tubes containing DMEM + 10% FBS and were immediately placed on ice and proceeded further for scRNA-seq analysis, as described below. In addition to the sorting of cells from WT embryos, cells were also sorted from GF transgenic Tg(*lck*:GFP) zebrafish to enrich *lck*^+^ cells for scRNA-seq. Fifty larvae were used as one replicate, and four replicates, that is, two hundred larvae in total, were used for each condition. The exposure, and the procedures for single-cell dissociation and DRAQ7 staining, were the same as for the WT zebrafish. However, the gating strategy was based on forward scatter, DRAQ7 and GFP. DRAQ7^−^GFP^+^ cells were sorted into tubes containing DMEM + 10% FBS and were immediately placed on ice, and processed for scRNA-seq analysis.

### Single-cell RNA sequencing

The samples were loaded on a 10x GemCode Single-Cell Instrument (10x Genomics) to generate single-cell gel beads in emulsion (GEMs), and libraries were constructed using the Chromium Next GEM Single Cell 3’ GEM, Library & Gel Bead Kit v3.1 (10x Genomics). Briefly, GEMs were generated by combining barcoded Single Cell 3’ v3.1 Gel Beads, a Master Mix containing cells and partitioning oil onto Chromium Next GEM Chip G. Following GEM generation, the gel bead was dissolved, primers containing an Illumina TruSeq Read 1, 10x Barcode, unique molecular identifier and poly(dT) sequence were released, and the cells were lysed. The barcoded, full-length cDNA was synthesized, purified and amplified by PCR for library construction. Dual-indexed libraries containing the P5 and P7 primers used in Illumina amplification were prepared for an estimated 5,000 nuclei per sample. Paired-end, dual indexing sequencing of libraries was conducted on a NovaSeq 6000 sequencing system (Illumina). Cell Ranger 6.0.1 (10x Genomics) pipelines (cellranger mkfastq and cellranger count) were used to convert Illumina Base call files to FASQT format, align sequencing reads to the zebrafish reference genome GRCz11 and generate feature-barcode matrices. The generated feature-barcode matrices were used for the subsequent analysis.

### Data analysis of 10x Genomics data

The analysis of the 10x Genomics data was performed using the Seurat toolkit (version 4.0.6) (available at https://satijalab.org/seurat/index.html) in the R environment (RStudio, version 4.2.0). A standard preprocessing workflow was applied, including QC metrics, data normalization and scaling, and the detection of highly variable features. Specifically, Chromium Single Cell 3’ samples with unique feature counts over 6,000 or less than 200 were filtered out. In addition, the percentage of mitochondrial content was set to be less than 10%. After QC, 3,115 single cells from the WT control sample and 3,012 single cells from the WT GO+BA sample (experiment 1) and 2,312 single cells from the Tg(*lck*:GFP) control sample and 2,669 single cells from the Tg(*lck*:GFP) GO+BA sample (experiment 2) were used for the downstream analysis. The raw counts that passed the QC were centred by a scale factor of 10,000 and log transformed. The highly variable features were detected using the ‘FindVariableFeatures’ command in Seurat by directly modelling the mean–variance relationship inherent in the single-cell data and 2,000 features per dataset were returned^45^. A linear transformation (scaling) was applied to the data before the principal component analysis. Only the previously determined highly variable features were used as the input for the calculation of principal components using the ‘RunPCA’ command in Seurat. The ‘JackStrawPlot’ and ‘ElbowPlot’ commands were applied to visualize the ranking of the principal components. Significant principal components showing strong enrichment of features with low *p* values were selected for the subsequent clustering analysis^46^. Specifically, 20 and 15 principal components were identified for the first and second RNA-seq experiment, respectively. The t-stochastic neighbour embedding transformation^47^ was achieved by the ‘RunTSNE’ command in Seurat. The positive marker genes in each cluster compared with all the remaining cells were identified using the ‘FindAllMarkers’ command. An identified feature requires to be detected at a minimum percentage of 0.25 in each of the cluster of cells and differentially expressed (on average) with a Log2FC threshold of 0.25 between the clusters. The cell clusters were annotated according to the Zebrafish Information Network (https://zfin.org/) and on the basis of current literature^25,48,49^. The visualization of the marker expression was demonstrated by heat maps, violin plots, feature plots and dot plots using the ‘DoHeatmap’, ‘VlnPlot’, ‘FeaturePlot’ and ‘DotPlot’ commands, respectively. The scRNA-seq integration analysis was further performed to identify the cell types that are present in both datasets and to find cell-type-specific responses to the stimulation^50^. The normalization and identification of variable features for each dataset were independently performed as described above. The features that were repeated variables across the datasets were selected for integration using the ‘SelectIntegrationFeatures’ command in Seurat, followed by ‘FindIntegrationAnchors’ and ‘IntegrateData’ commands to create an integrated data assay. The standard workflow described above, including scaling data, principal component analysis, clustering and t-stochastic neighbour embedding transformation was also performed on the integrated data. The ‘split.by’ argument was used to visualize the two conditions side by side, and the ‘subset’ argument was used to plot the data in a specific cluster. The transcriptomics data from both analyses are deposited in ArrayExpress at EMBL-EBI.

### Statistical analysis

Experiments were performed at least three times, each in triplicate for each condition. Statistical differences were analysed using the Student’s t-test (GraphPad Prism version 8.2.0). The data presented are mean values ± standard deviation (s.d.). The differences between groups or treatments were considered significant when *p* < 0.05. The analysis of the 16S rRNA data and scRNA-seq data is described above.

### Reporting summary

Further information on research design is available in the Nature Portfolio Reporting Summary linked to this article.

## Online content

Any methods, additional references, Nature Portfolio reporting summaries, source data, extended data, supplementary information, acknowledgements, peer review information; details of author contributions and competing interests; and statements of data and code availability are available at 10.1038/s41565-022-01260-8.

## Supplementary information

Supplementary Information Supplementary Figs. 1–17 and Tables 1–3.

Reporting Summary.

Supplementary Data 1 Statistical source data for Supplementary Fig. 2.

Supplementary Data 2 Statistical source data for Supplementary Fig. 4.

Supplementary Data 3 Statistical source data for Supplementary Fig. 7.

Supplementary Data 4 Statistical source data for Supplementary Fig. 10.

## Data Availability

The 16S rRNA gene sequencing data are deposited at NCBI (accession no. PRJNA682318) and the two scRNA-seq datasets are deposited at ArrayExpress (accession nos. E-MTAB-11984 and E-MTAB-11991). Source data are provided with this paper.

## Source data

Source Data Fig. 1 Statistical source data.
Source Data Fig. 2 Statistical source data.
Source Data Fig. 3 Statistical source data.
