# Supplementary material for: Graphene oxide elicits microbiome-dependent type 2 immune responses via the aryl hydrocarbon receptor
Source: Nat Nanotechnol. 2022 Dec 12;18(1):42–8. doi: 10.1038/s41565-022-01260-8 (PMC9879769; doi:10.1038/s41565-022-01260-8)
Supplement: Supplementary file 1 — Supplementary Figs. 1–17 and Tables 1–3. [file 41565_2022_1260_MOESM1_ESM.pdf]

nature nanotechnology

Letter

https://doi.org/10.1038/s41565-022-01260-8

Graphene oxide elicits microbiome-dependent type 2 immune responses via the aryl hydrocarbon receptor

In the format provided by the
authors and unedited

SUPPORTING INFORMATION

Graphene oxide elicits microbiome-dependent type 2 immune responses *via* the aryl hydrocarbon receptor

G. Peng, H. Sinkko, H. Alenius, N. Lozano,
K. Kostarelos, L. Bräutigam, and B. Fadeel*

**Corresponding author. Institute of Environmental Medicine, Nobels väg 13, Karolinska Institutet, 171 77 Stockholm, Sweden; E-mail: bengt.fadeel@ki.se*

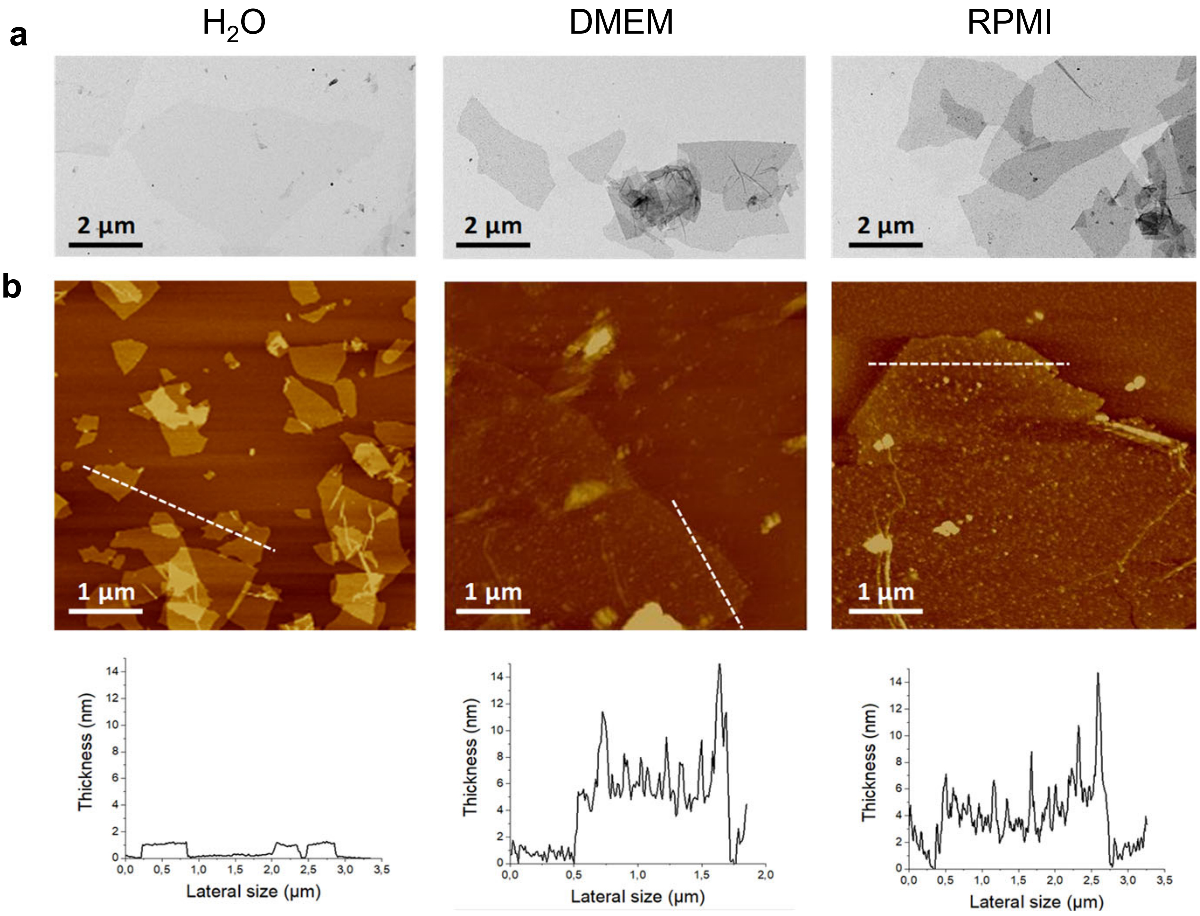

Figure S1. Physicochemical characterization of GO in water, DMEM and RPMI media. (a) Representative TEM images. (b) Representative AFM images and thickness analysis of GO. Cross-section analysis highlighted by the line in the height image. The structural properties of GO sheets dispersed in the E3 medium were reported previously by us [Peng G., et al. *Nanoscale*. 2020;12(32):16730-7].

Figure S1

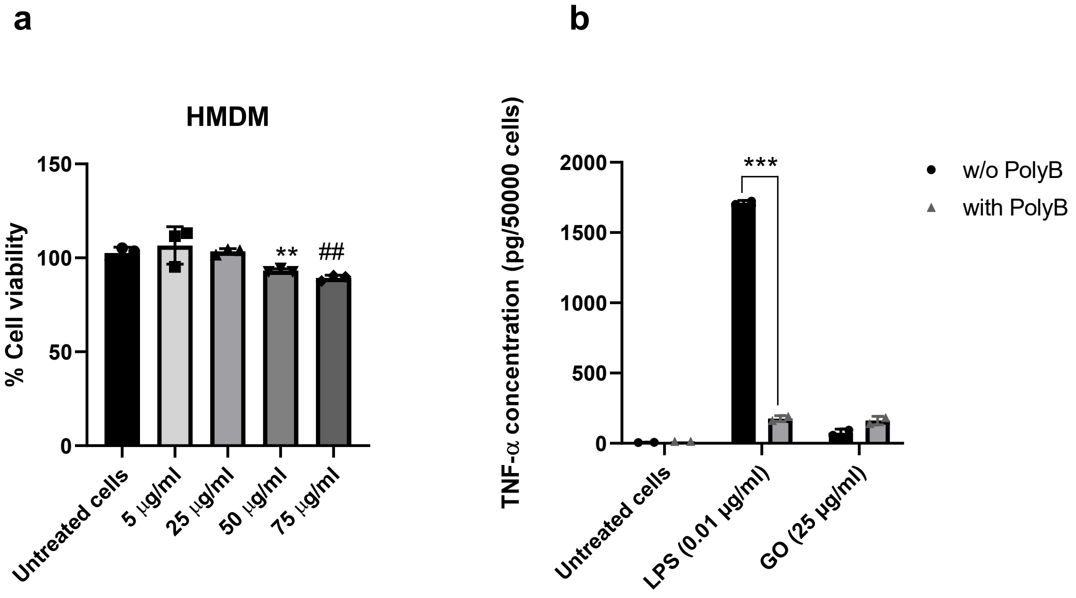

Figure S2. The endotoxin content of the GO samples was evaluated using the macrophage-based TNF-α expression test (TET) [Mukherjee S.P., et al. *PLoS One*. 2016;11(11):e0166816]. (a) Cytotoxicity assessment by Alamar Blue assay in primary human monocyte-derived macrophages (HMDM) following exposure to GO for 24 h. Data are presented as mean values ± S.D. of three independent experiments (n=3). Student's *t*-test (two-sided) was used to determine significant differences (** *p*=0.0087; ## *p*=0.0031). (b) HMDM were exposed to 25 μg/mL of GO in the presence and absence of polymyxin B and TNF-α secretion was quantified by ELISA. Data are presented as mean values ± S.D. of three independent experiments (n=3). Student's *t*-test (two-sided) was used to determine significant differences (*** *p*=0.00014).

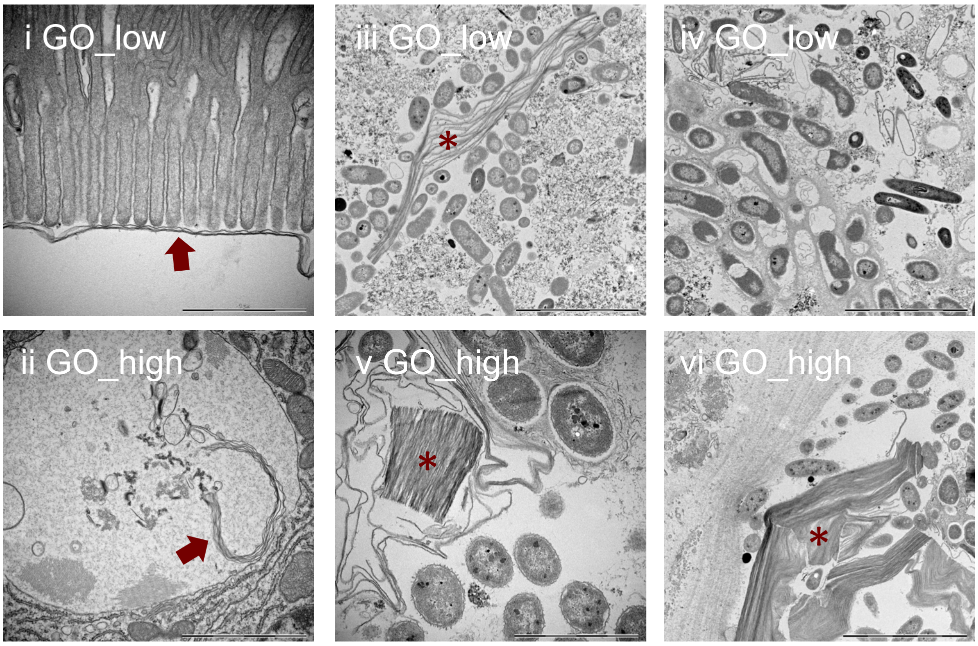

Figure S3. GO interaction with gastrointestinal cells and with gut-resident bacteria visualized by TEM analysis. The red arrows indicate GO sheets in close apposition to microvilli (i) and GO internalized by gastrointestinal epithelial cells (ii). The stars in panels (iii), (v), and (vi) mark bundles of GO sheets in the lumen of the GI-tract. The fish were exposed to a low or high dose of GO for 7 days as indicated. Scale bar: 1 μm (panel i), 2 μm (panels ii and v), and 5 μm (panels iii, iv, vi).

Figure S3

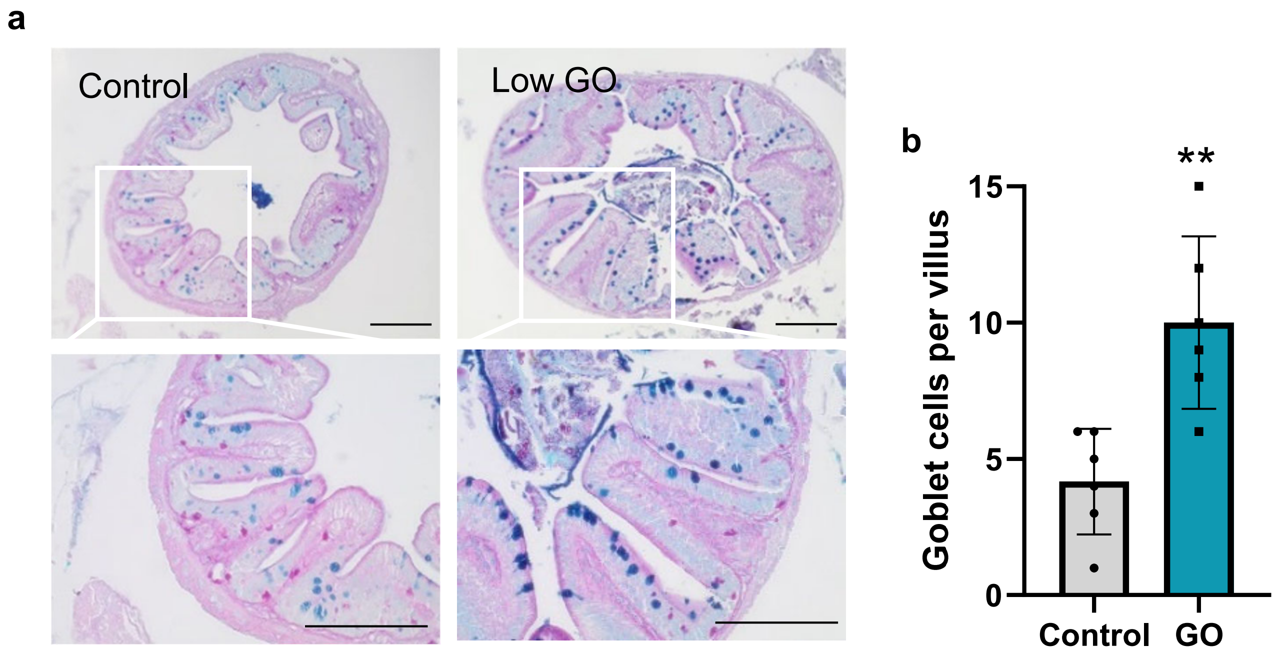

Figure S4. Goblet cell hyperplasia in zebrafish intestines upon low dose (50 μg/L) GO exposure. Goblet cells were identified with AB-PAS staining (a) and the number of goblet cells *per* villus were counted (b) as detailed in Methods. Scale bars: 100 μm. The results are shown as mean values ± S.D. of six slices per condition (n=6). Student's *t*-test (two-sided) was used for the analysis of comparisons between control and the treatment (** p=0.0032).

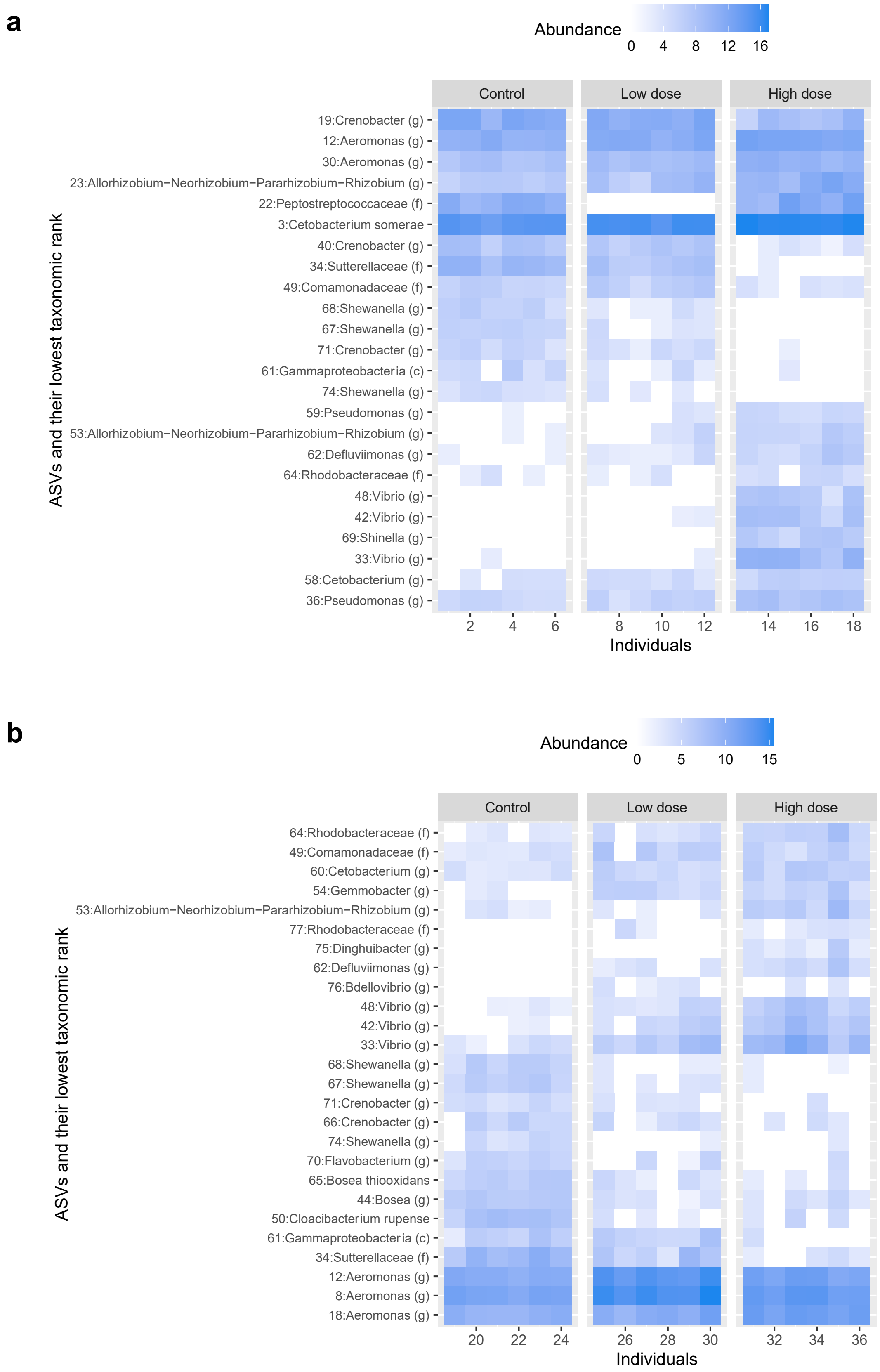

Figure S5. Gut microbiome analysis in adult zebrafish. Significantly different bacteria identified among wild-type (a) and *ahr2*$^{+/-}$ fish (b). The abundance of amplicon sequence variants (ASVs) were normalized, log2 transformed, and assigned to their lowest taxonomic rank.

Figure S5

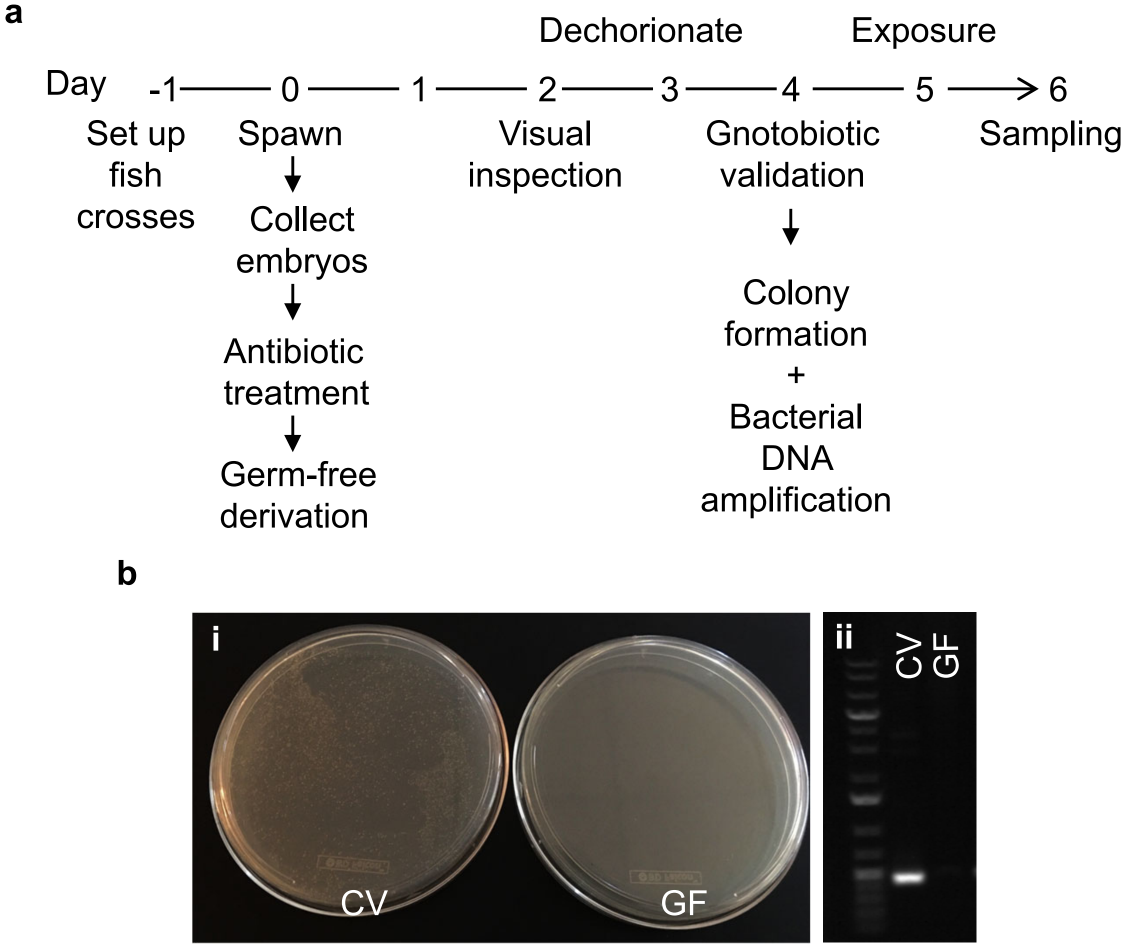

Figure S6. Generation and validation of germ-free zebrafish embryos. (a) Overview of procedures for germ-free zebrafish derivation (adapted from: Pham L.N., et al. *Nat Protoc.* 2008;3(12):1862-75). (b) Germ-free zebrafish validation as evidenced by (i) the absence of zebrafish homogenate-induced bacterial colony formation on LB plates, and (ii) the absence of bacterial DNA amplification by PCR. CV, conventional; GF, germ-free.

Figure S6

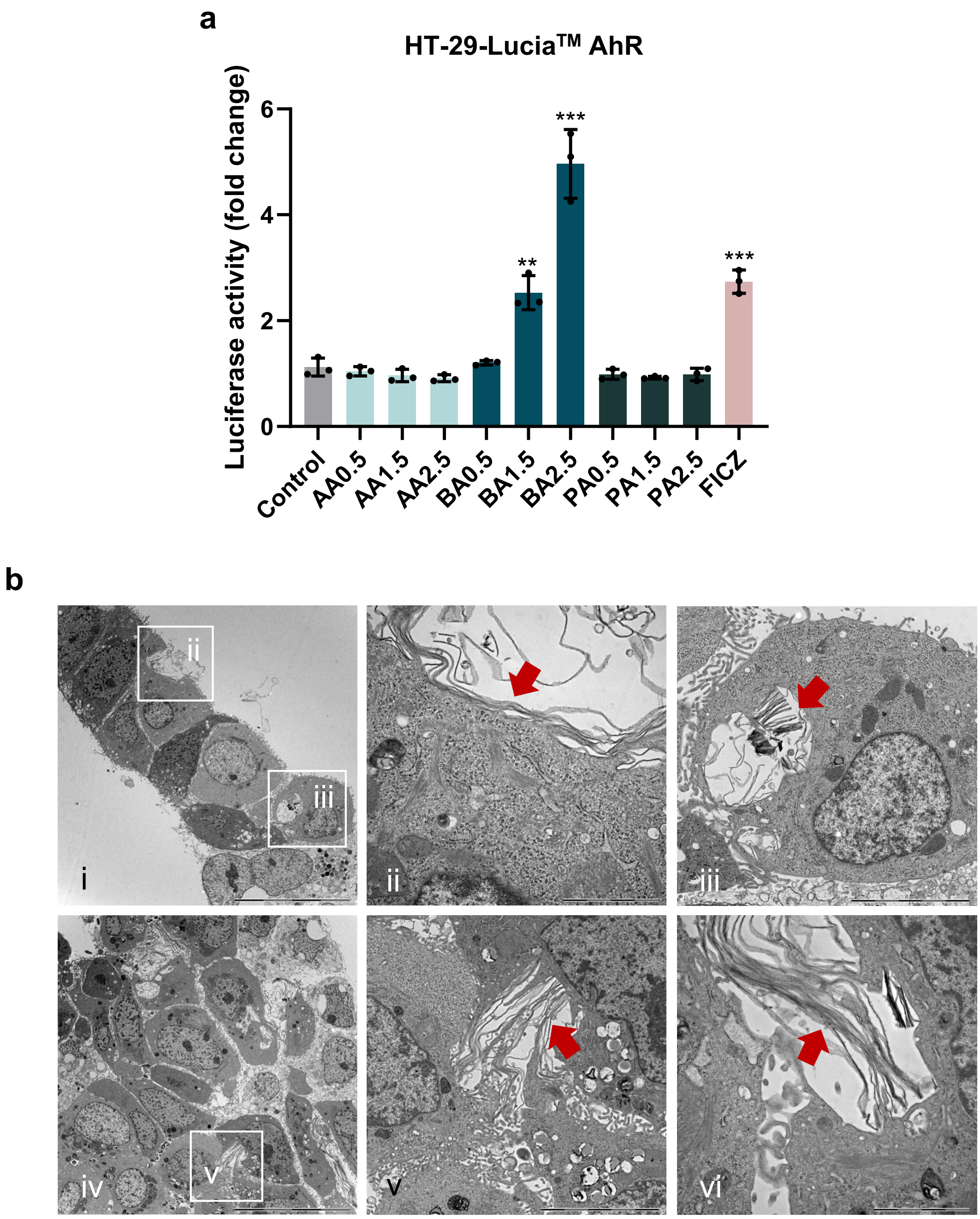

Figure S7. The microbial short-chain fatty acid butyrate (BA) acts as a ligand for AhR in gastrointestinal cells. (a) AhR activity in the human HT29-Lucia™ AhR reporter cell line. AhR activity was evidenced at 24 h in cells exposed to BA, but not to other SCFAs (AA and PA). FICZ was used as a positive control. Data are expressed as fold responses compared to non-induced cells. Results are mean values ± S.D. of three independent experiments (n=3). Student's *t*-test (two-sided) was used to determine significant differences (control vs. BA1.5 *p*=0.0026; control vs. BA2.5 *p*=0.0006; control vs. FICZ *p*=0.0006, ***p*<0.01, ****p*<0.001). (b) TEM analysis of differentiated HT-29 cells exposed to GO. The differentiated cells were found to develop structures resembling microvilli. The red arrows indicate GO sheets close to these microvilli (ii, v), and bundles of GO sheets internalized in the cells (iii, vi). Scale bars: 20 µm (i, iv); 5 µm (iii, v); 2 µm (ii, vi).

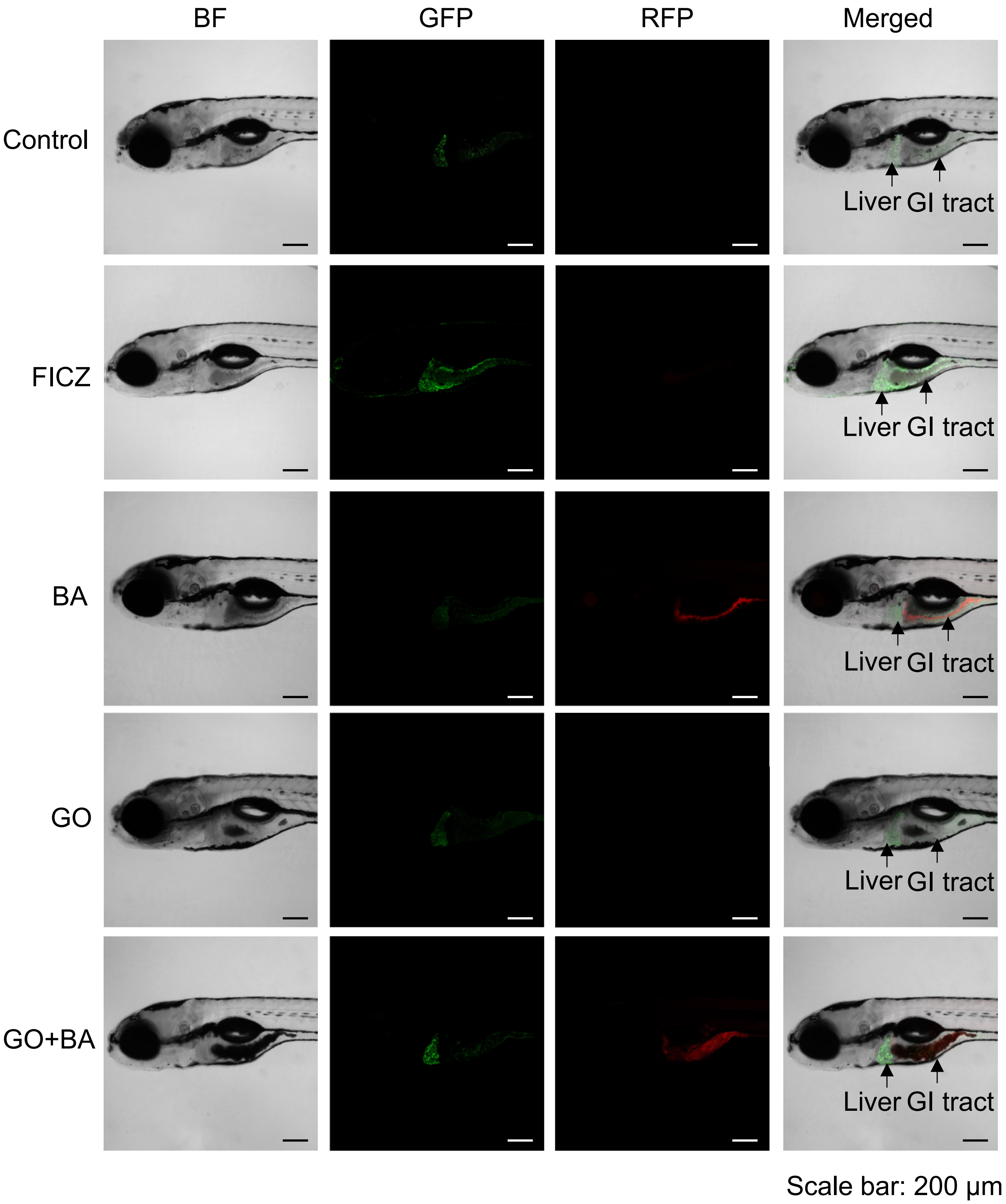

Figure S8. AhR activation triggered by GO+BA. To study AhR activation, the *Tg(cyp1a:GFP)* reporter line was used under germ-free (GF) conditions. Exposure to FICZ (200 nM) for 24 h resulted in a strong induction of *cyp1a* in the liver and gastrointestinal (GI) tract. Fluorescent resorufin butyrate (BA) (5 μM) was found to accumulate in the GI-tract with a modest induction of *cyp1a*. In contrast, prominent *cyp1a* activation was observed in the GO+BA group.

Figure S8

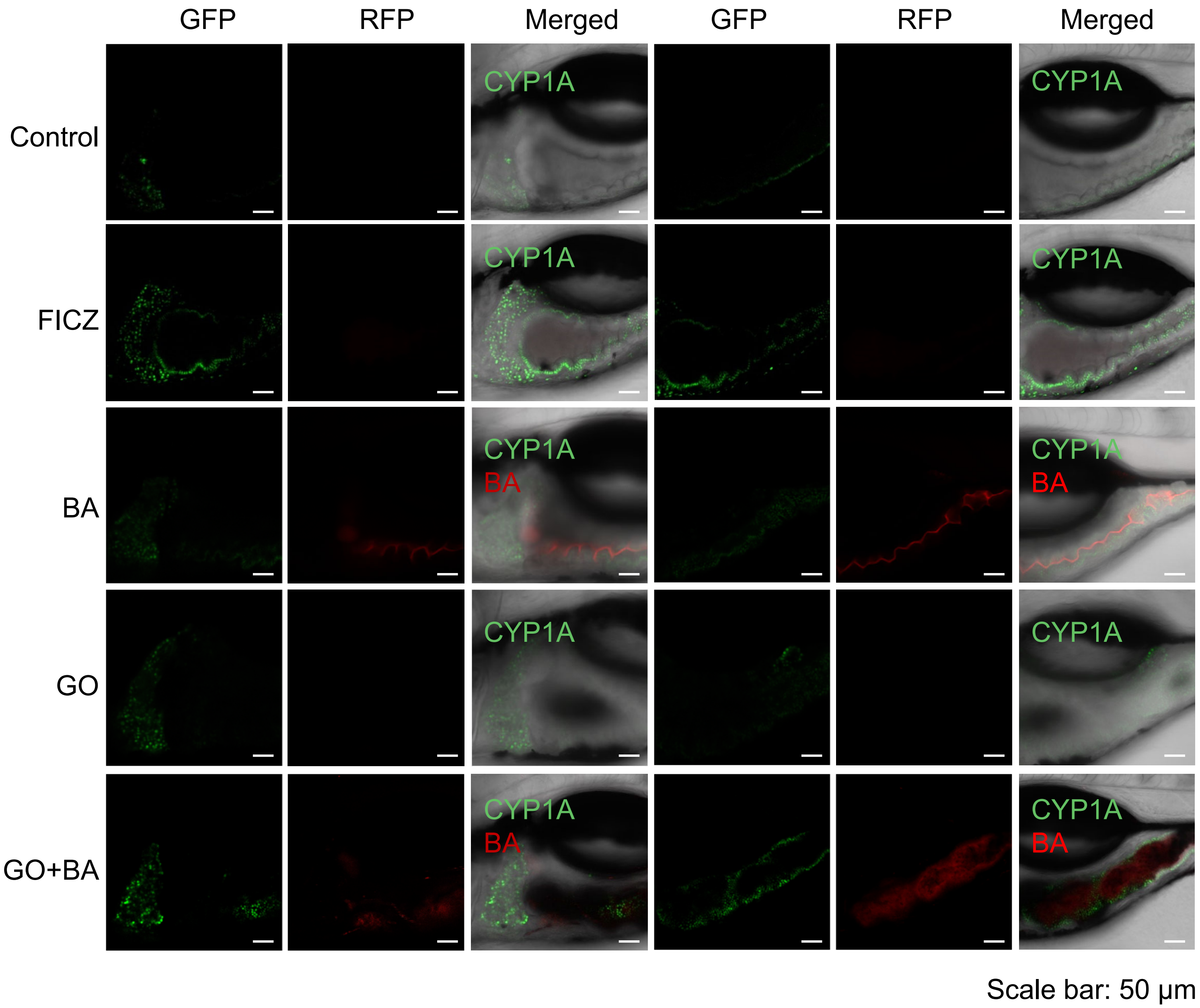

Figure S9. AhR activation triggered by GO+BA under GF conditions in the *Tg(cyp1a:GFP)* reporter line. The data shown are the magnified confocal images of the fish shown in Figure S8.

Figure S9

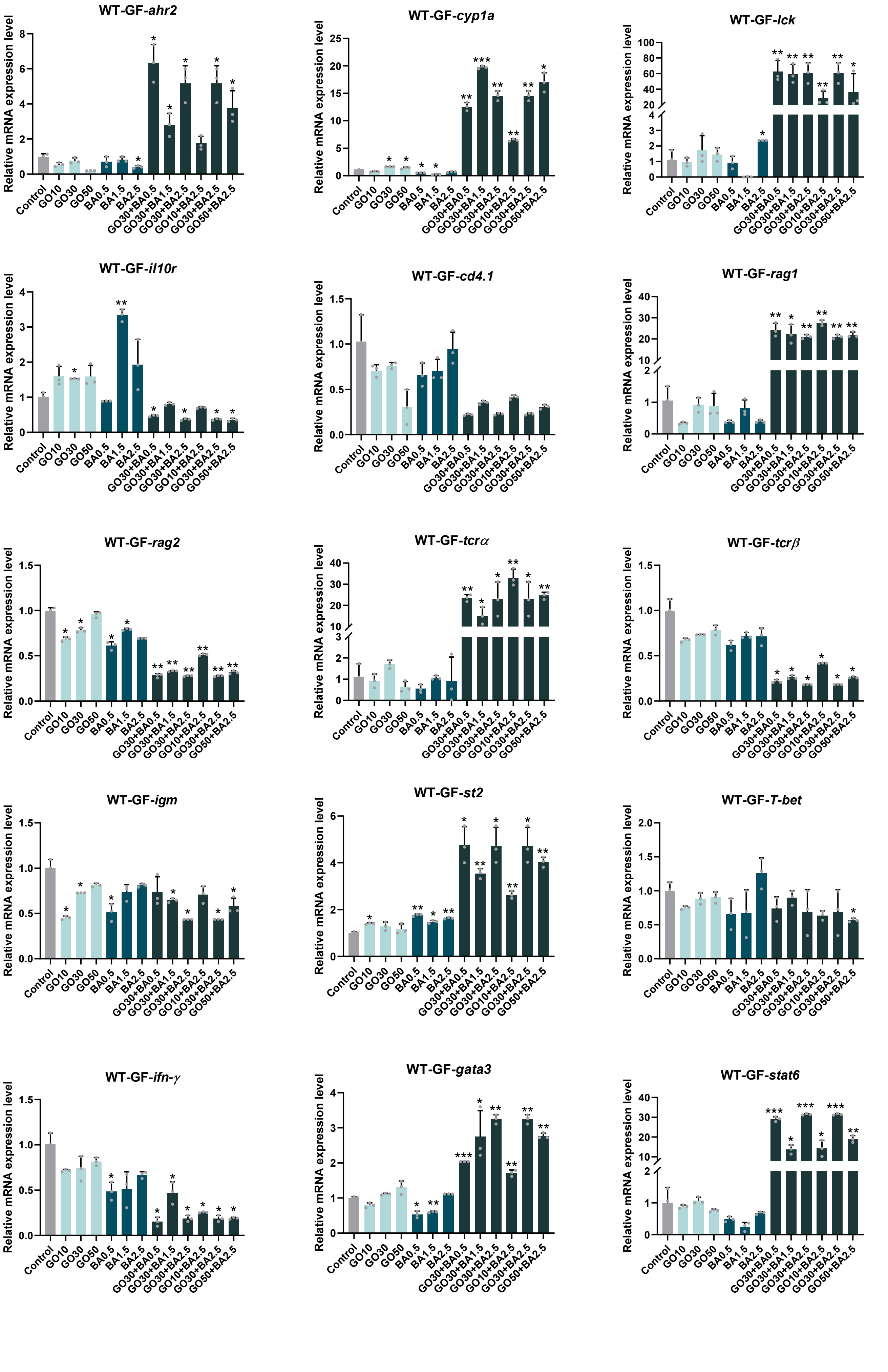
WT-GF-ahr2
WT-GF-cyp1a
WT-GF-lck
WT-GF-il10r
WT-GF-cd4.1
WT-GF-rag1
WT-GF-rag2
WT-GF-tcrα
WT-GF-tcrβ
WT-GF-igm
WT-GF-st2
WT-GF-T-bet
WT-GF-ifn-γ
WT-GF-gata3
WT-GF-stat6
Relative mRNA expression level
Control
GO10
GO30
GO50
BA0.5
BA1.5
BA2.5
GO30+BA0.5
GO30+BA1.5
GO30+BA2.5
GO10+BA2.5
GO30+BA2.5
GO50+BA2.5

Figure S10

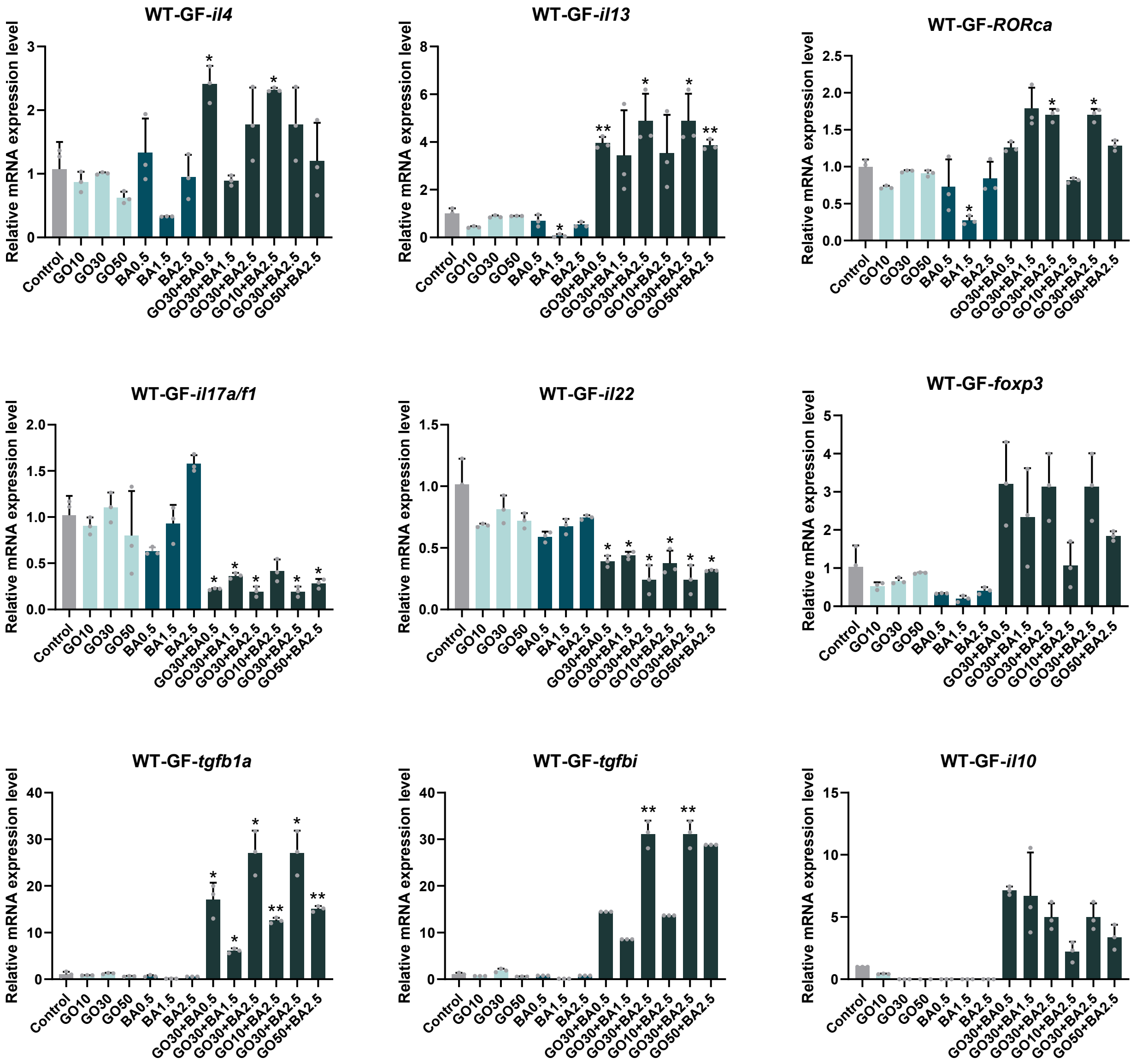

Figure S10. GO+BA impact on immune cell population markers in germ-free zebrafish. Experiments were performed using wild-type (WT) zebrafish larvae maintained under germ-free (GF) conditions. The embryos were thus exposed to GO alone, BA alone, or to a combination of GO plus BA at the indicated concentrations. RNA was extracted from whole embryos as described in Methods and PCR analysis was performed in order to evaluate the expression of selected genes related to AhR activation and genes corresponding to different lymphocyte populations. Note that the panel with “WT-GF-*lck*“ results is also shown in Figure 3b. Note also that the gene denoted here as *st2* is also known as *il1rl1*. Data are presented as mean values ± S.D. of three independent experiments (n=3). Student’s *t*-test (two-sided) was used to analyze the differences between control and exposures. * $p<0.05$, ** $p<0.01$, *** $p<0.001$.

Figure S10

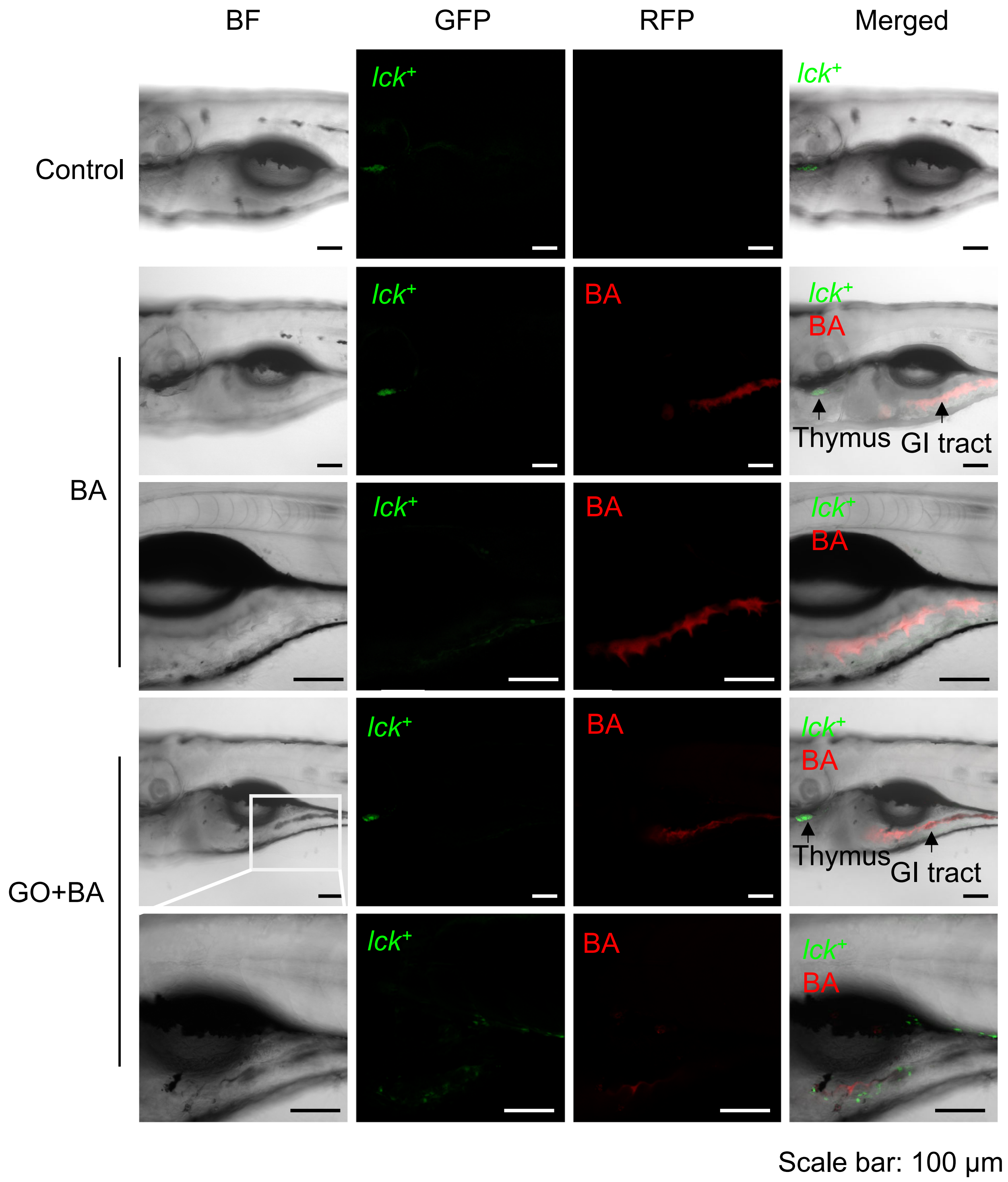

Figure S11. Visualization of *lck*$^+$ induction and homing using the *Tg(lck:GFP)* reporter line under germ-free (GF) conditions. Fluorescent resorufin butyrate (red) (5 µM) was used for these experiments. BA was found to accumulate in the GI-tract, while *lck*$^+$ cells (green) were found in the thymus. However, exposure to GO+BA triggered the homing of *lck*$^+$ cells to the GI tract of GF fish.

Figure S11

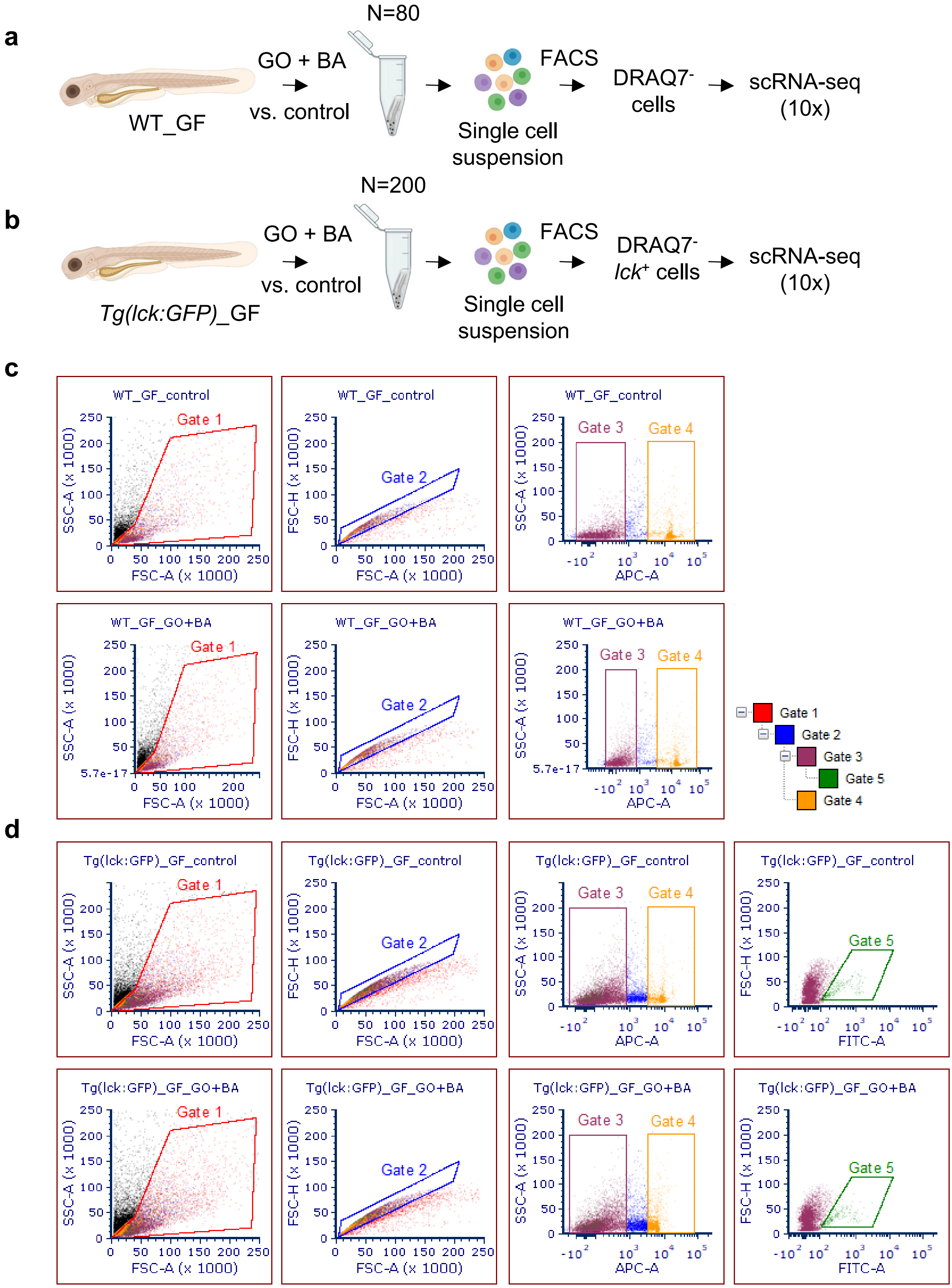

Figure S12. Sample preparation for scRNA-seq. (a,b) Schematic figures show the experimental design and the sample preparation for scRNA-seq. Following germ-free (GF) derivation, zebrafish embryos (5 dpf) were exposed or not to GO+BA. Briefly, single cell suspensions were obtained through enzymatic dissociation and mechanical pipetting, optimized to maintain the cell viability. The cells were then labeled with DRAQ7 and sorted by FACS to collect viable cells; the first experiment was thus performed on cells derived from whole embryos. For the second experiment, GF *Tg(lck:GFP)* embryos were used and cells were sorted on lck, followed by RNA sequencing of the lck^{+} cells. (c,d) Cell population discrimination by flow cytometry. For wild-type GF embryos, the cells in gate 3 (DRAQ7^{-}) were sorted for scRNA-seq (c); for *Tg(lck:GFP)* GF embryos, the cells in gate 5 (DRAQ7^{-}lck^{+}) were sorted for scRNA-seq analysis (d).

Figure S12

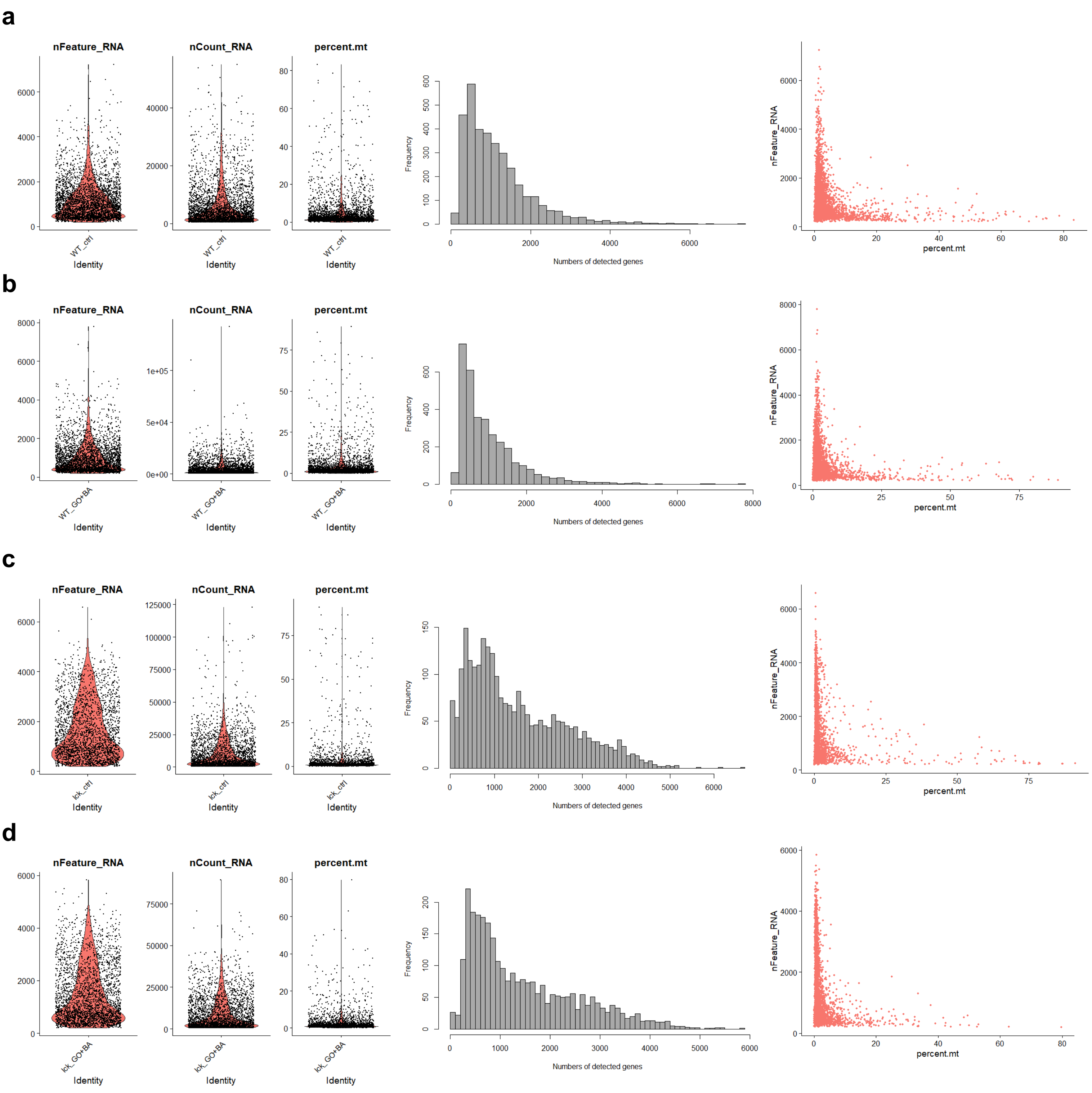

Figure S13. Quality control (QC) of the 10x sequencing data. (a) WT_control, (b) WT_GO+BA, (c) *Tg(lck)*_control, (d) *Tg(lck)*_GO+BA. Shown from left to right are the numbers of detected genes, mRNA, and percent mitochondrial content in single cells from each sample, followed by histograms for the numbers of detected genes, and scatter plots with the number of detected genes *versus* the percent mitochondrial content. Cells with unique feature counts over 6000 or less than 200 as well as those with >10% mitochondrial counts were filtered out. The total features and numbers of cells in each sample that passed the QC were: (a) WT_control: 20,501 genes, 3115 cells, (b) WT_GO+BA: 19,866 genes, 3012 cells, (c) *Tg(lck)*_control: 19,000 genes, 2312 cells, (d) *Tg(lck)*_GO+BA: 18,504 genes, 2669 cells.

Figure S13

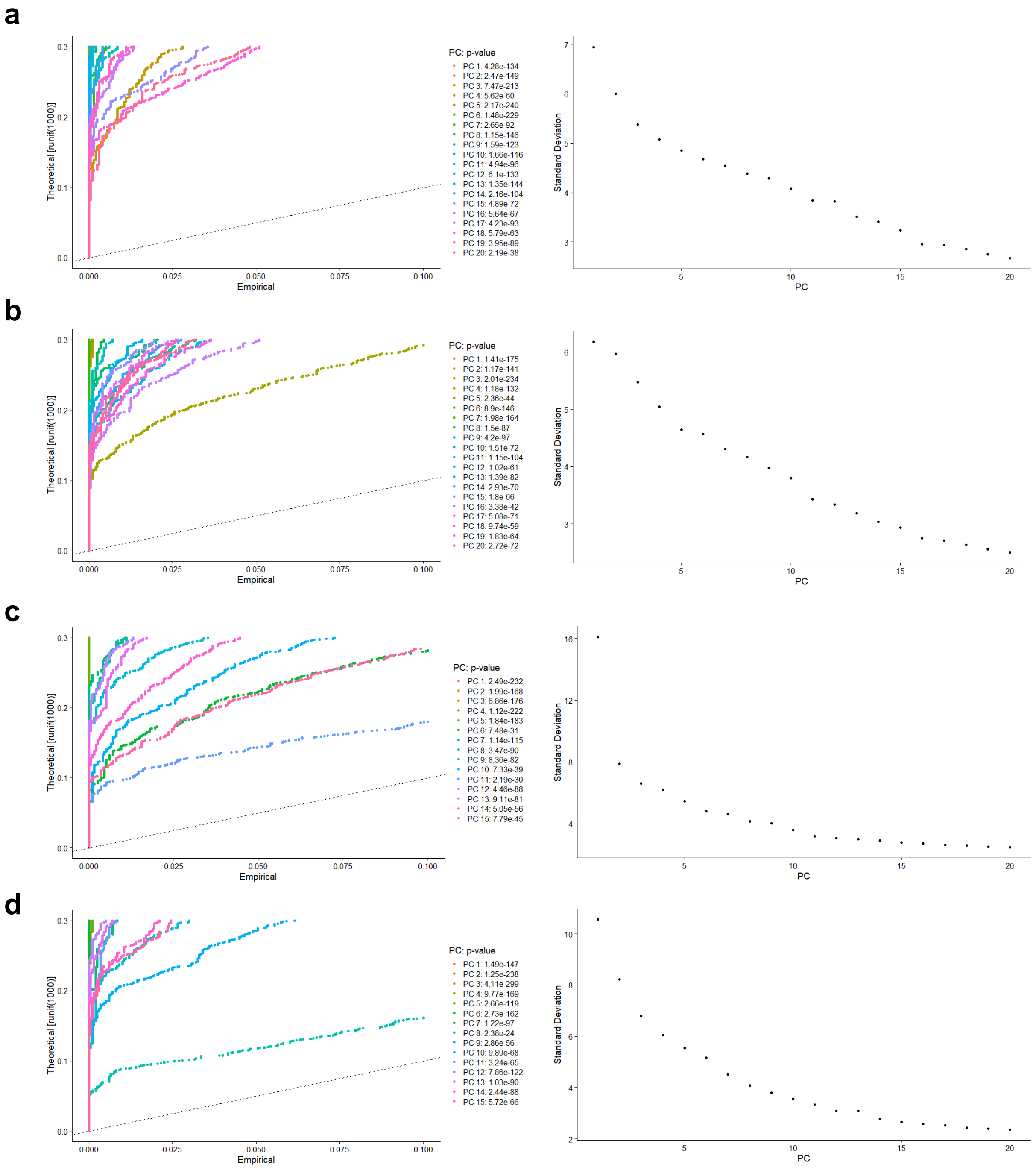

Figure S14. The dimensionality of the datasets was determined using the Seurat JackStraw function: (a) WT_control, (b) WT_GO+BA, (c) *Tg(lck)*_control, (d) *Tg(lck)*_GO+BA. For the WT samples (a,b), the first 20 PCs were significant with low *p* values (left), and the elbow plots showed an 'elbow' around PC20 (right), suggesting that the majority of true signals were captured in the first 20 PCs. Hence, 20 PCs were selected for the following cluster and non-linear dimensional reduction. Similarly, 15 PCs were selected for the lck-sorted samples (c,d) for the cluster and non-linear dimensional reduction.

Figure S14

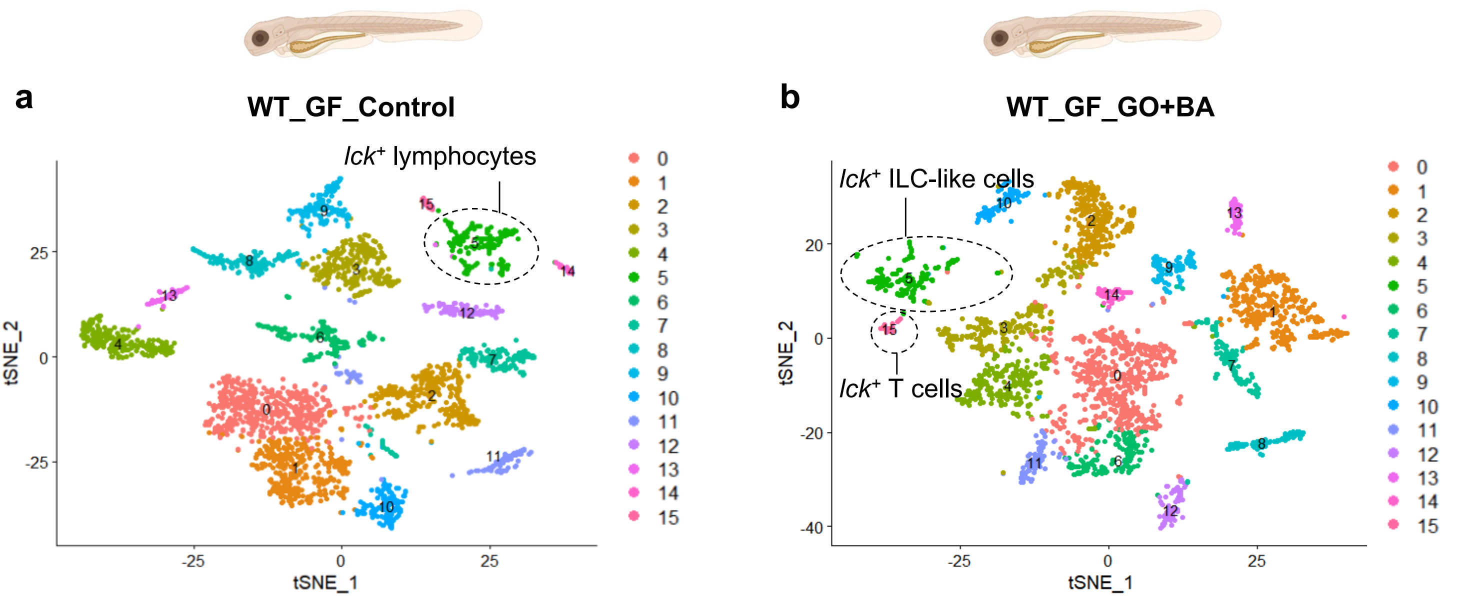

Figure S15. scRNA-seq analysis of wild-type (WT) germ-free (GF) embryos. (a) The 2D projection of the tSNE analysis showing the *lck*$^+$ lymphocytes (cluster 5) in control fish. (b) The 2D projection of the tSNE analysis showing the emergence of two separate *lck*$^+$ clusters in fish exposed to GO+BA, *i.e.*, *lck*$^+$ ILCs-like cells (defined as *nitr*$^+$*rag1*$^-$) (cluster 5) and *lck*$^+$ T cells (defined as *nitr*$^-$*rag1*$^+$) (cluster 15). On the following page, feature plots corresponding to genes expressed in cluster 5 (*lck*$^+$ lymphocytes) in control fish (c) and genes expressed in cluster 5 (*lck*$^+$ ILCs-like cells) and cluster 15 (*lck*$^+$ T cells) in fish exposed to GO+BA (d) are shown.

Figure S15

c

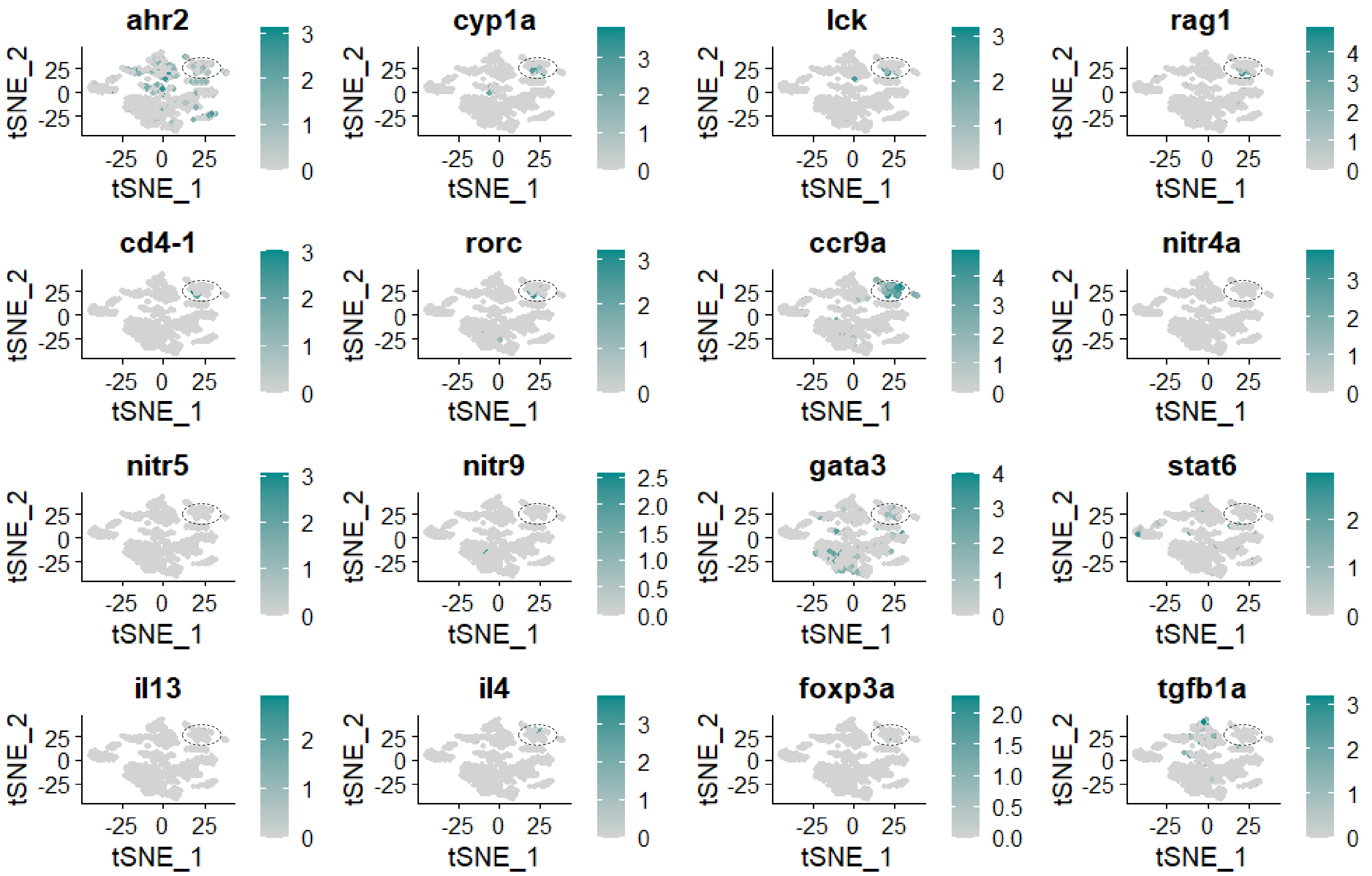

d

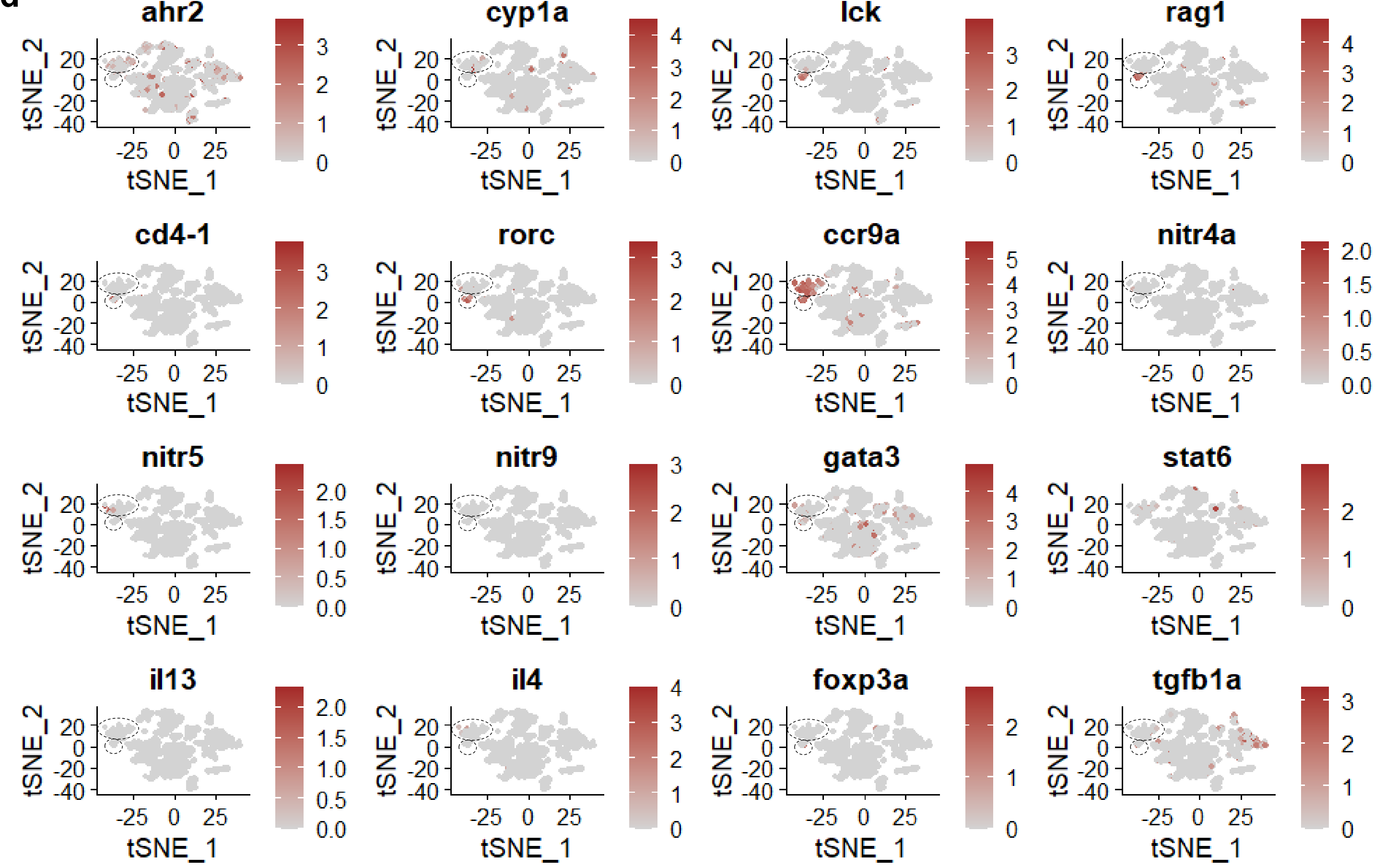

Figure S15

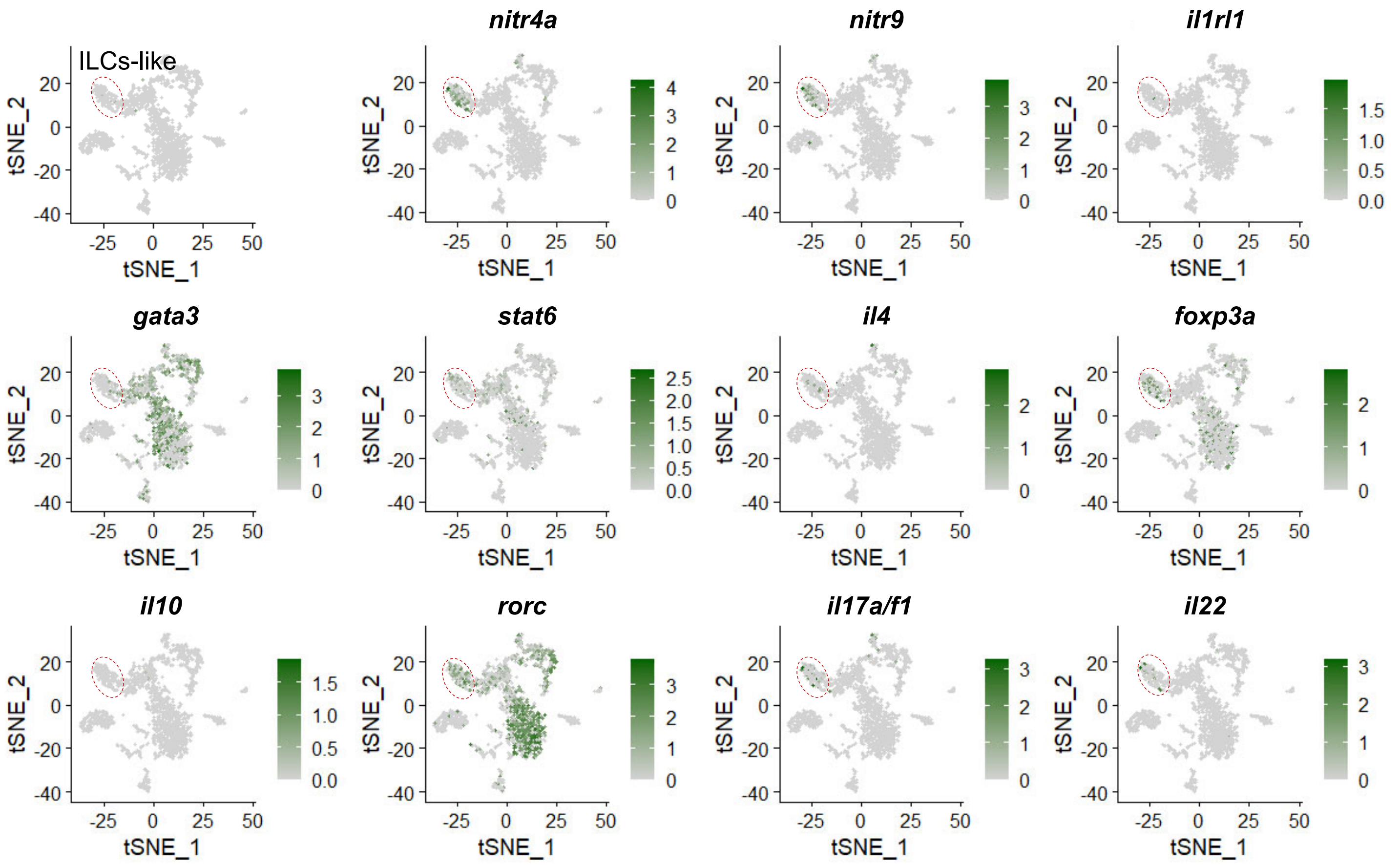

Figure S16. Feature plots of the ILC-like cell markers in the germ-free (GF) *Tg(lck:GFP)* control sample (refer to Figure 4a). The corresponding feature plots for the GF GO+BA sample are shown in Figure 4e. Note that the gene encoding IL-10 is not expressed in cluster 4 in control cells (above) while the same gene is expressed in the ILC-like cluster in GO+BA fish.

Figure S16

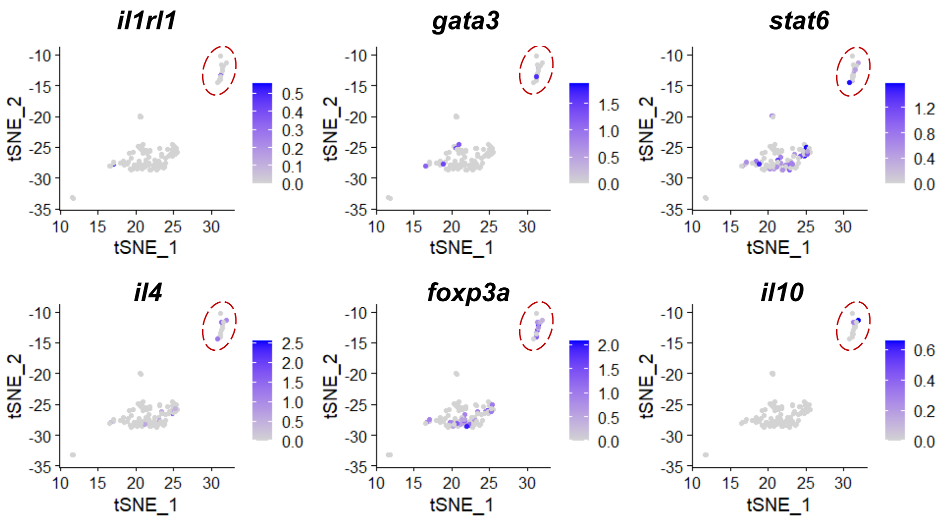

Figure S17. Feature plots of the ILC-like cell population (cluster 8) in the germ-free (GF) *Tg(lck:GFP)* sample exposed to GO+BA. The data shown are the same as in Figure 4e, but the other cell clusters were removed to focus specifically on cluster 8 and on genes related to the $ILC2_{10}$-like cell population (*i.e.*, cells expressing ILC2 cell markers along with *foxp3* and *il10*).

Table S1. The overall differences in zebrafish gut microbiota between genotypes and exposures. PERMANOVA was performed separately for genotypes and exposures using between-sample distances calculated for normalized abundances of the ASVs. Statistical significances are based on 9999 random permutations (refer to Methods).

Between genotypes						
	WT		*ahr2*$^{+/-}$			
Variable	R^2	*P* value	R^2	*P* value		
Exposure	0.46	0.0006	0.34	0.0018		
Gender	0.02	0.5394	0.02	0.7876		
Exposure:Gender	0.12	0.1224	0.09	0.4892		
Residuals	0.39		0.55			
Between exposures						
	Control		Low dose[a]		High dose[a]	
Variable	R^2	*P* value	R^2	*P* value	R^2	*P* value
Genotype	0.65	0.0031	0.32	0.0293	0.21	0.0532
Gender	0.03	0.4227	0.07	0.3872	0.21	0.055
Genotype:Gender	0	0.8727	0.05	0.4741	0.01	0.9447
Residuals	0.32		0.56		0.58	

[a]Low dose: 50 μg/L; high dose: 500 μg/L (continuous exposure for 7 days).

Table S2. Differences in zebrafish microbiota upon GO exposure within genotypes (WT vs. AhR-deficient). Pairwise comparisons performed by PERMANOVA which used Bray-Curtis between-sample distance and normalized abundances of the ASVs. Statistical significances are based on 9999 random permutations (refer to Methods).

WT	*Comparison*	R^2	*P* value	*P* value adjusted
	Control <-> High dose[a]	0.58	0.004	0.012
	Control <-> Low dose[a]	0.12	0.2867	0.2867
	High dose <-> Low dose	0.35	0.019	0.0285
ahr2$^{+/-}$	*Comparison*	R^2	*P* value	*P* value adjusted
	Control <-> High dose	0.36	0.004	0.012
	Control <-> Low dose	0.29	0.015	0.0225
	High dose <-> Low dose	0.19	0.0839	0.0839

[a]Low dose: 50 μg/L; high dose: 500 μg/L (continuous exposure for 7 days).

Table S3. Primer sequences used in this study (zebrafish larvae).

Gene	Primer sequences
ahr2	F: ATAATGACAGTTCCCACACT<br>R: ATGAAGTAGGCTTGGAGTAA
cyp1a	F: TAAATTCATGAAGAGGCTGG<br>R: TGTTGTCCTTATCGAACGTA
lck	F: GCCGAAGAAGATCTCGATGGT<br>R: TCCCCATGTTTACGTATTTTGTC
il10r	F: AATGTAACTGTTCTGTGGGA<br>R: GTTCCATTTAGCATGAGGAC
cd4.1	F: AAGAGTTGAGAAAGCTCCAGTG<br>R: CTGGTCTTGCGTCGTCTGTA
rag1	F: AATGATGCAAGGCAGAGGA<br>R: CAATGATGCCCACATCCC
rag2	F: TGAGACTCAGAAGCGCATGG<br>R: ACCAAGTACGACTGTGGCTG
tcrα	F: GAAGCCGAATATTTACCAAGTG<br>R: AACAAACGCCTGTCTCCT
tcrβ	F: AAATCAACAAACAAATTCACCTG<br>R: TATGCCAGCTTCATCCACTG
igm	F: GTTTCCTCAGCTCAACCA<br>R: AGTATAATCTCCTTCCTTCCC
st2	F: AGTAAGAACGGAGGCTTCGC<br>R: CGTCCATAGCTGCACAGTGA
tbet	F: ACACTGGCACTCACTGGATG<br>R: CTCCTTCACCTCCACGATGT
ifnγ	F: AAGATTCTCAGCTACATAATGCACACC<br>R: ATGCTCATCAGTAGATTCTGCTCAC
gata3	F: GCTTCTTCCTCCTCGCTGTC<br>R: TGCACTCTTTGTCTTCCTGTCG
stat6	F: CGGTAGTCAGGAAATCAATGC<br>R: ATCTGTCCAATAGTCTCGTAGG
il4	F: CATCCAGAGTGTGAATGGGA<br>R: TTCCAGTCCCGGTATATGCT
il13	F: GAAGTGTGAGCATGATTATTTC<br>R: CTCGTCTTGGTGGTTGTAAG
rorca	F: CGTGATGCGGTGAAGTTTGG<br>R: CCTGGGACTGCTGGTGCTT
il17a/f1	F: CATTCGGTGCTGAGGGGG<br>R: AGCCGGTATGAATGATCTGC
il22	F: CACGAAATGAAGACGATCAC<br>R: CTTCTTCTTGCAGCATATCG
foxp3	F: GCAACCAGCCTTTTCCACAAGC<br>R: GACTATATGGATGCTTCCCAGTA
tgfb1a	F: AGACCTGCTGTATGCGCAAGCTTTAC<br>R: ACCATGTTGGACAATTGCTCCACCTTG
tgfbi	F: CGCTGACCTCAACAAACTCATGAGAG<br>R: TGGTCACTCACAATTTTAGGAGGCAG
il10	F: TTTCCTTTAAGACTGAGGGAG<br>R: CATCCATAGGGACTGTTTATG
rpl13	F: GCACCAAACTCATCATCATCTTC<br>R: AAAGCCTTGAAGTTCTTCTC
