# Supplementary material for: Graphene oxide elicits microbiome-dependent type 2 immune responses via the aryl hydrocarbon receptor
Source: Nat Nanotechnol. 2022 Dec 12;18(1):42–8. doi: 10.1038/s41565-022-01260-8 (PMC9879769; doi:10.1038/s41565-022-01260-8)
Supplement: Supplementary file 2 — Reporting Summary. [file 41565_2022_1260_MOESM2_ESM.pdf]

nature research

Corresponding author(s): Prof. Bengt Fadeel, Karolinska Institutet

Last updated by author(s): Sep 30, 2022

Reporting Summary

Nature Research wishes to improve the reproducibility of the work that we publish. This form provides structure for consistency and transparency in reporting. For further information on Nature Research policies, see our Editorial Policies and the Editorial Policy Checklist.

Statistics

For all statistical analyses, confirm that the following items are present in the figure legend, table legend, main text, or Methods section.

n/a	Confirmed	
☐	☒	The exact sample size (*n*) for each experimental group/condition, given as a discrete number and unit of measurement
☐	☒	A statement on whether measurements were taken from distinct samples or whether the same sample was measured repeatedly
☐	☒	The statistical test(s) used AND whether they are one- or two-sided<br>*Only common tests should be described solely by name; describe more complex techniques in the Methods section.*
☐	☒	A description of all covariates tested
☐	☒	A description of any assumptions or corrections, such as tests of normality and adjustment for multiple comparisons
☐	☒	A full description of the statistical parameters including central tendency (e.g. means) or other basic estimates (e.g. regression coefficient) AND variation (e.g. standard deviation) or associated estimates of uncertainty (e.g. confidence intervals)
☐	☒	For null hypothesis testing, the test statistic (e.g. *F*, *t*, *r*) with confidence intervals, effect sizes, degrees of freedom and *P* value noted<br>*Give P values as exact values whenever suitable.*
☒	☐	For Bayesian analysis, information on the choice of priors and Markov chain Monte Carlo settings
☒	☐	For hierarchical and complex designs, identification of the appropriate level for tests and full reporting of outcomes
☒	☐	Estimates of effect sizes (e.g. Cohen's *d*, Pearson's *r*), indicating how they were calculated

Our web collection on statistics for biologists contains articles on many of the points above.

Software and code

Policy information about availability of computer code

Data collection	MiSeq Control Software v. 4.0 was used to collect 16S rRNA gene sequencing data from intestines of adult zebrafish. QuantStudio5 Real-Time PCR System Software was used to collect gene expression data, i.e., RT-PCR data from zebrafish larvae. NovaSeq Control Software was used to collect the single-cell RNA sequencing data (10x Genomics). BD FACSDiva 9.0.1 was used to collect the fluorescence-activated cell sorting data.
Data analysis	16S rRNA gene sequence analysis was performed with the following software/code: cutadapt (version 2.9), FastQC (version v0.11.9), multiqc (version 1.9.dev0), and analyses were performed in the R environment (3.6.2, R Core Team, 2019) using DADA2 package (version 1.14.1) with rRNA gene database Silva (version 138). Further statistical analyses for 16S rRNA gene sequence data were performed using R packages vegan and metagenomeSeq. Single-cell RNA sequencing analysis was pre-processed using Cell Ranger pipelines (cellranger mkfastq and cellranger count, version 6.0.1, 10x Genomics), and further analyzed using the Seurat (version 4.0.6) in the R environment (RStudio, version 4.2.0). FACS data was analysed using FCS Express™ v. 7.0 software (DeNovo Software, Pasadena, CA). ZEN (version 3.0) software including the 2.5D view tool (for the analysis of the confocal microscopy data) along with Fiji (ImageJ) and GraphPad Prism (version 8.2.0) were also used in this study.

For manuscripts utilizing custom algorithms or software that are central to the research but not yet described in published literature, software must be made available to editors and reviewers. We strongly encourage code deposition in a community repository (e.g. GitHub). See the Nature Research guidelines for submitting code & software for further information.

Data

Policy information about availability of data

All manuscripts must include a data availability statement. This statement should provide the following information, where applicable:

- Accession codes, unique identifiers, or web links for publicly available datasets
- A list of figures that have associated raw data
- A description of any restrictions on data availability

The scRNA-seq data (using whole zebrafish embryos and lck-sorted cells from zebrafish embryos) are deposited at ArrayExpress (accession no. E-MTAB-11984 and E-MTAB-11991, respectively) and the 16S rRNA gene sequencing data obtained in adult fish are deposited at NCBI (accession no: PRJNA682318). The zebrafish reference genome GRCz11 can be assessed at NCBI (GCF_000002035.6). Source data for Figures 1-3 and for Supplementary Figures S2, S4, S7, and S10 are provided.

Field-specific reporting

Please select the one below that is the best fit for your research. If you are not sure, read the appropriate sections before making your selection.

☒ Life sciences ☐ Behavioural & social sciences ☐ Ecological, evolutionary & environmental sciences

For a reference copy of the document with all sections, see nature.com/documents/nr-reporting-summary-flat.pdf

Life sciences study design

All studies must disclose on these points even when the disclosure is negative.

Sample size	No statistical method was used to predetermine the sample size. For single-cell RNA sequencing experiments, each condition was performed with four pooled replicates and each replicate contained twenty (for the study using whole wild-type embryos) and 50 larvae (for the study using sorted cells from Tg(lck:GFP) embryos), which generated enough viable single cells for the sequencing. For other experiments using zebrafish larvae, each condition was performed in three replicates and each replicate sample contained ten larvae. Ten larvae generated sufficient total RNA for the RT-qPCR analysis. For experiments using adult zebrafish, three female and three male individuals were used in each genotype for each experiment. For the human cell lines, each condition was performed in triplicate in three independent experiments.
Data exclusions	For gene expression analysis in the ahr2-/- zebrafish, the data were collected from four individuals, two female and two male. The exclusion criteria are specified in the text (in short, some individuals were excluded due to poor survival in the ahr2-/- group exposed to high dose GO).
Replication	All attempts at data replication were successful as reported in the main text and figure legends and figures (and refer to Methods for details).
Randomization	For the zebrafish experiments (larvae and adult fish), the animals in each genotype were randomly allocated into experimental groups. For experiments using the human cell line, different cell passages were used for each biological replicate (denoted as independent experiments).
Blinding	The zebrafish with different genotypes were collected separately. The investigators were not blinded to group allocation. The 16S rRNA gene sequencing data and single-cell transcriptomics data are not affected by knowledge of sample identities (for statistical analysis, see Methods).

Reporting for specific materials, systems and methods

We require information from authors about some types of materials, experimental systems and methods used in many studies. Here, indicate whether each material, system or method listed is relevant to your study. If you are not sure if a list item applies to your research, read the appropriate section before selecting a response.

Materials & experimental systems

n/a	Involved in the study	
☒	☐	Antibodies
☐	☒	Eukaryotic cell lines
☒	☐	Palaeontology and archaeology
☐	☒	Animals and other organisms
☒	☐	Human research participants
☒	☐	Clinical data
☒	☐	Dual use research of concern

Methods

n/a	Involved in the study	
☒	☐	ChIP-seq
☐	☒	Flow cytometry
☒	☐	MRI-based neuroimaging

Eukaryotic cell lines

Policy information about cell lines

Cell line source(s)	Human colon adenocarcinoma cell line HT-29 from Sigma-Aldrich, and HT-29-Lucia™ AhR reporter cell line from InVivoGen.

Authentication	The HT-29 cell line was authenticated by the supplier using STR-PCR profiling. Reporter activity of the reporter cell line has been verified (validated) by functional assays by the supplier; refer to validation results reported by the supplier InVivoGen.
Mycoplasma contamination	MycoAlert™ Mycoplasma Detection Kit (Lonza) was used to screen for mycoplasma contamination. Cell lines thus tested negative for mycoplasma contamination. The cell lines were regularly screened and at no time was mycoplasma detected.
Commonly misidentified lines (See ICLAC register)	No commonly misidentified cell lines (i.e., through cross-contamination or other mechanisms, e.g., mislabeling) were used.

Animals and other organisms

Policy information about studies involving animals; ARRIVE guidelines recommended for reporting animal research

Laboratory animals	Zebrafish (Danio rerio) were used in the current study including four different genotypes, i.e., wild-type (WT) AB strain (KI Zebrafish Core Facility), AhR deficient strain (ahr2hu3335) (Wellcome Sanger Institute, UK), CYP1A reporter strain (Tg(cyp1a:GFP) (China Zebrafish Resource Center), and LCK reporter strain (Tg(lck:GFP) (European Zebrafish Resource Center). For adult zebrafish, both female and male individuals were used at 4.5 months. For studies on zebrafish larvae, 5 day-post-fertilization (dpf) larvae were used.
Wild animals	The study did not involve the use of wild animals. Experiments were conducted at the Zebrafish Core Facility at Karolinska Institutet.
Field-collected samples	The study did not involve samples collected in the field. Experiments performed at the Zebrafish Core Facility at Karolinska Institutet.
Ethics oversight	The zebrafish study was approved by the Regional Committee for Animal Experiments in Stockholm (ethical permit no. 14049-2019).

Note that full information on the approval of the study protocol must also be provided in the manuscript.

Flow Cytometry

Plots

Confirm that:

☒ The axis labels state the marker and fluorochrome used (e.g. CD4-FITC).

☒ The axis scales are clearly visible. Include numbers along axes only for bottom left plot of group (a 'group' is an analysis of identical markers).

☒ All plots are contour plots with outliers or pseudocolor plots.

☒ A numerical value for number of cells or percentage (with statistics) is provided.

Methodology

Sample preparation	The single cell suspensions were prepared from whole zebrafish larvae by enzymatic dissociation and mechanical pipetting as specified in the methods. The cell suspension was then stained with a fluorescent DNA dye DRAQ7 to exclude non-viable cells (Invitrogen Cat# D15106) at the dose of 3 µM for 10 min at room temperature before the fluorescence-activated cell sorting.
Instrument	BD FACSAria III, BD Biosciences, NJ, USA. Experiments were performed at the Biomedicum Flow Cytometry Core Facilty at KI.
Software	BD FACSDiva 9.0.1 was used to collect data. Data were analyzed using FCS Express™ v. 7.0 (DeNovo Software, Pasadena, CA).
Cell population abundance	For wild-type zebrafish, the percentage of viable cells collected was around 24% while for Tg(lck:GFP) zebrafish samples, the percentage of viable, GFP (high) positive cells collected was around 0.5%. The cell viability of the obtained cells was checked with trypan blue staining (Bio-Rad Laboratories), and the single cell suspensions were inspected under the light microscope.
Gating strategy	For wild-type, germ-free zebrafish samples, the gating strategy was based on the forward scatter and DRAQ7 as shown in the Supporting Information. Thus, the DRAQ7 negative (viable) cells were sorted for subsequent single-cell RNA sequencing. For the germ-free Tg(lck:GFP) zebrafish samples, the gating strategy was based on forward scatter, DRAQ7, and GFP (see Supporting Information). DRAQ7 negative, GFP positive cells were sorted for downstream single-cell RNA sequencing analysis.

☒ Tick this box to confirm that a figure exemplifying the gating strategy is provided in the Supplementary Information.
